# Diversification in immunogenicity genes caused by selective pressures in invasive meningococci

**DOI:** 10.1099/mgen.0.000422

**Published:** 2020-08-10

**Authors:** Philip H.C. Kremer, John A. Lees, Bart Ferwerda, Merijn W. Bijlsma, Neil MacAlasdair, Arie van der Ende, Matthijs C. Brouwer, Stephen D. Bentley, Diederik van de Beek

**Affiliations:** ^1^​ Amsterdam UMC, University of Amsterdam, Department of Neurology, Amsterdam Neuroscienc, Amsterdam, The Netherlands; ^2^​ Parasites and Microbes, Wellcome Sanger Institute, Hixton, Cambridge, UK; ^3^​ MRC Centre for Global Infectious Disease Analysis, Department of Infectious Disease Epidemiology, Imperial College London, London, UK; ^4^​ Amsterdam UMC, Department of Medical Microbiology and the Netherlands Reference Laboratory for Bacterial Meningitis, Amsterdam, The Netherlands

**Keywords:** 4CMenB, antigens, evolution, genome sequencing, *Neisseria meningitidis*

## Abstract

We studied population genomics of 486 *Neisseria meningitidis* isolates causing meningitis in the Netherlands during the period 1979–2003 and 2006–2013 using whole-genome sequencing to evaluate the impact of a hyperendemic period of serogroup B invasive disease. The majority of serogroup B isolates belonged to ST-41/44 (41 %) and ST-32 complex (16 %). Comparing the time periods, before and after the decline of serogroup B invasive disease, there was a decrease of ST-41/44 complex sequences (*P*=0.002). We observed the expansion of a sub-lineage within ST-41/44 complex sequences being associated with isolation from the 1979–2003 time period (*P*=0.014). Isolates belonging to this sub-lineage expansion within ST-41/44 complex were marked by four antigen allele variants. Presence of these allele variants was associated with isolation from the 1979–2003 time period after correction for multiple testing (Wald test, *P*=0.0043 for FetA 1–5; *P*=0.0035 for FHbp 14; *P*=0.012 for PorA 7–2.4 and *P*=0.0031 for NHBA two peptide allele). These sequences were associated with 4CMenB vaccine coverage (Fisher’s exact test, *P*<0.001). Outside of the sub-lineage expansion, isolates with markedly lower levels of predicted vaccine coverage clustered in phylogenetic groups showing a trend towards isolation in the 2006–2013 time period (*P*=0.08). In conclusion, we show the emergence and decline of a sub-lineage expansion within ST-41/44 complex isolates concurrent with a hyperendemic period in meningococcal meningitis. The expansion was marked by specific antigen peptide allele combinations. We observed preliminary evidence for decreasing 4CMenB vaccine coverage in the post-hyperendemic period.

## Data Summary

The authors confirm all supporting data, accession numbers, code and protocols have been provided within the article or through supplementary data files.

**Impact Statement**

In the last decade of the previous century there was a high incidence of *N. meningitidis* meningitis in the Netherlands. This primarily involved one subset of *N. meningitidis* bacteria. In this study we compared the genomes from meningococci, causing bacterial meningitis, isolated from 1979 to 2003 to those isolated from 2006 to 2013. We find that within the subset of bacteria causing the high incidence, there is a further subdivision of bacteria, which all have the same types of proteins on the outside, which interact with the human immune system (antigens). We also find that the combination of these antigens on the outside of *N. meningitidis* are changing over time. This is important, as vaccines are available targeting various of these antigens, while not others. Our analyses hint at the fact that the antigens targeted by the current vaccines are becoming increasingly uncommon over time. Our results show the importance of monitoring the composition of the types of antigens in this bacterial species closely.

## Introduction

*Neisseria meningitidis* (the meningococcus) is an important cause of meningitis, severe sepsis and septic shock, which are associated with high morbidity and mortality rates [1]. The epidemiology of invasive meningococcal disease in The Netherlands has been well established [2]. For serogroup B disease, a hyperendemic period started in 1982 and had a peak incidence rate of 3.43 per 100 000 population in 1993. This rate steeply decreased from 2002 onwards to 0.39 per 100 000 population in 2012. This decline co-occurred with a serogroup C epidemic for which vaccination was introduced in 2002 [2].

Antigen allele variants cluster in lineages and clades within the phylogeny of meningococcal populations [3, 4]. *N. meningitidis* has various antigens that interact with the host immune system [5]. *N. meningitidis* outer membrane proteins and polysaccharide structures are continuously changing under the selective pressure from the human immune system [6, 7]. Of these, the capsule polysaccharide, outer membrane protein (PorA), ferric enterobactin receptor A (FetA), Neisseria Heparin-Binding Antigen (NHBA) and factor H binding protein (FHbp) are of major importance [5, 8], because they are antigens in existing serogroup B vaccines (Bexsero, Trumemba) or in serogroup B vaccines under development (PorA-based vesicle vaccine, Nonamen [9], FetA-based vaccine [10] or FHbp-based vesicle vaccine [11].

4CMenB (Bexsero) is a vaccine against serogroup B isolates that has been developed based on four bacterial surface proteins, the antigens PorA, NHBA, FHbp and Neisseria Adhesin A (NadA). It is currently implemented in the National Immunization Program of the UK, Spain, Southern Australia and Italy. Coverage for this vaccine depends on the structural integrity of these antigens, which have high genetic variability compared to the capsule polysaccharide of single serogroups [12].

We used whole-genome sequencing to compare genomic differences in serogroup B isolates causing meningitis from the hyperendemic period with serogroup B isolates from a later period in time. We determined frequency and type of antigen alleles, which we correlated to putative selection pressures and predicted vaccine coverage.

## Methods

### Meningococcal isolates

Isolates cultured from cerebrospinal fluid (CSF) from adults aged 16 years or older with community-acquired bacterial meningitis from three nationwide prospective studies from 1993 to 2003 and 2006 to 2013 were included in this study [13–15]. These isolates were supplemented with a random subset of samples from 1979 to 1993 from the collection of the Netherlands Reference Laboratory for Bacterial Meningitis (NRLBM), for which a blood and cerebrospinal fluid paired sample was available as part of a previous study [16] During the inclusion periods, notification for this disease was mandatory. Patients or their legal representatives gave written informed consent for participation. Patients with hospital-acquired bacterial meningitis, neurosurgical procedures, or those within 1 month following neurosurgical procedure or neurotrauma were excluded. The Medical Ethics Committee of the Amsterdam University Medical Center, location AMC, University of Amsterdam, the Netherlands, approved the studies.

### Bacterial whole-genome sequencing

DNA extraction from isolate cultures and subsequently WGS sequencing as described earlier [16]. Briefly, DNA from *N. meningitidis* strains was extracted using the Maxwell RSC Cultured Cells DNA kit according to the manufacturer’s protocol (Promega, Madison, WI, USA). Sequencing was performed using multiplexed libraries on the Illumina HiSeq platform to produce paired-end reads of 100 nucleotides in length (Illumina, San Diego, CA, USA). Quality control involved analysis of contamination, number and length of contigs, GC content and N50 parameter. Sequences for which one or more of these quality-control parameters deviated by more than three standard deviations from the mean, were excluded. Sequences of the bacterial samples were assembled using a standard assembly pipeline [17]. The median number of contigs was 85 (range 54–133), median GC content 53.83% (range 53.43–54.00%), average genome length 2 160 459 (range 2066672–2 389 876), and median coverage 204-fold (interquartile range 193–216). Serogroups and sequence types were determined from the whole-genome sequence by in-house scripts. Alleles for immunogenicity genes (*porA*, *fetA, NHBA* and *fHbp*) were determined from whole-genome sequences. Clonal complexes were determined from sequence types.

### Data availability

Fastq sequences of bacterial isolates were deposited in the European Nucleotide Archive (ENA, accession numbers in Table S1, available in the online version of this article). Metadata as serogroup, clonal complex, antigen peptide alleles and year of isolation have been added as Table S2.

### Pan-genome generation and phylogenetic tree

Genome sequences were annotated with PROKKA, version 1.11 [18]. Roary (version 3.5.0) with default parameters was used to extract clusters of orthologous genes, referred to as gene groups, and create a core gene alignment at a sequence identity threshold of 95% [19]. This process identified a pan-genome of 7530 gene groups and a core genome (shared by 100% of strains) of 1175 (Fig. S1). A maximum-likelihood phylogeny of single-nucleotide polymorphisms (SNPs) in the core genome of all sequenced isolates was produced with RAxML (version 7.8.6) assuming a general time reversible model of nucleotide substitution with a γ-distributed rate heterogeneity [20]. Trees and metadata were visualized with Microreact [21].

### Recombination purged phylogeny

A Bayesian clustering algorithm (hierBAPS, version 8.13, with parameters: 3 clustering iterations and 18 maximum number of clusters) was used to determine clusters within the phylogeny [22]. These sequence clusters are subsequently referred to as clades. To remove recombination events from core genomes, isolates from three clades were mapped against a randomly selected reference from the same clade with SMALT (version 0.7.4, using default parameters), creating a pseudoalignment. A phylogenetic tree for each clade was generated by running RAxML version 7.8.6 on these pseudoalignments [20]. The whole-genome pseudoalignments and trees were used as input to infer areas of increased SNP density and thereby determine recombination to mutation ratios with Gubbins (version 1.7.4, default parameters) [23]. These areas of increased SNP density were removed in order to obtain sequences without recombination regions, which were then used to infer recombination corrected phylogenetic trees for each cluster.

### Mutation rate, temporal correlation and molecular clock

Genetic distances within recombination purged trees for each clade were correlated to the sampling dates of corresponding samples with BactDating (version 1.0.6) to determine the correlation coefficient [24]. The validity of a molecular clock assumption was checked for each clade. Sequences in these clades and their sampling dates were used to infer the mutation rate in sites per year. A Mann–Whitney U test was performed in R to determine whether the mutation rate differed significantly. The date of emergence for the most common recent ancestor for nodes on the tree for each clade were determined with BactDating (MCMC was run for 1.0e6 iterations) [24]. To determine selective pressures on antigen genes, sequences were aligned with Clustal Omega (version 1.2.1), gene trees inferred with IQ-TREE (version 1.6.10, assuming a general time reversible model plus invariable sites with γ-distributed rate heterogeneity and fast tree search) [25] and d*N*/d*S* was calculated with HyPhy (version 2.5, default parameters) [26].

### Frequency of antigen alleles

From the whole-genome sequence data, *porA*, *fetA*, *nhbA* and *fHbp* gene sequences were identified by blast matches and compared to the PubMLST database for *Neisseria* species to determine peptide alleles (https://pubmlst.org/neisseria/ accessed December 2019). Determination of the FHbp peptide allele was not possible for two sequences because the *fHbp* gene was on a contig break. The *fetA* gene was missing from the sequence of one isolate. The PorA peptide allele was partially determined for 12 sequences, of which nine sequences had an undeterminable VR1 domain and three sequences an undeterminable VR1 and VR2 domain. The *nhba* gene was not fully sequenced in seven isolates. To determine the association of antigen peptide alleles per time period, logistic regression followed by the Wald test in R was performed (version 3.6.1). Antigen peptide alleles were categorized in two nominal categories; the most prevalent and ‘other’ category. Cut-off for statistical significance was a *P* value of 0.05, after correction for multiple testing.

### Vaccine coverage

We predicted 4CMenB (Bexsero, GSK) vaccine coverage based on PorA, NHBA and FHbp peptide alleles as described elsewhere [27]. Consistent with gMATS, Neisseria Adhesin A (NadA) was not considered [27]. Vaccine coverage was defined as predicted coverage for one of the peptide alleles. No vaccine coverage was defined as no predicted coverage for any of the peptide alleles. All other cases were determined as unpredictable. Fisher’s exact test in R was done to determine differences in vaccine coverage compared to no or unpredictable coverage between groups.

## Results

### Bacterial whole-genome sequencing reveals a sub-lineage expansion associated with serogroup B hyperendemic period

**Fig. 1.**
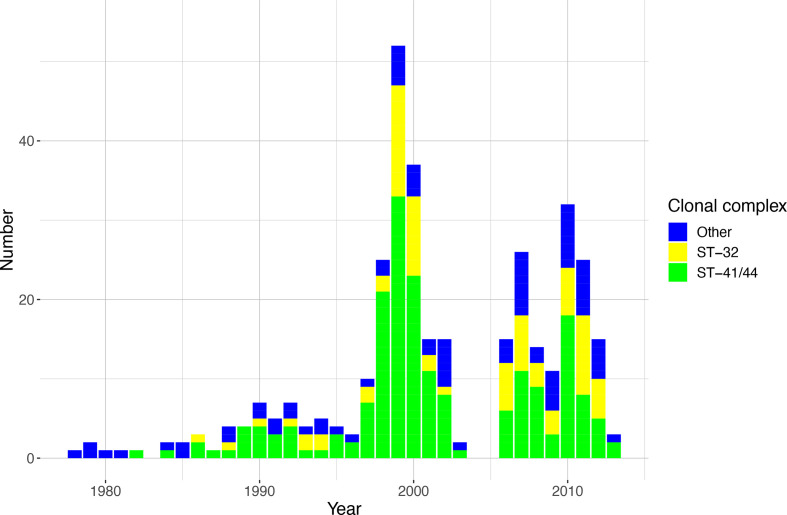
The proportion of ST-41/44 complex sequences isolated from the 1979–2003 time period is higher than the proportion isolated from 2006 to 2013.

**Fig. 2.**
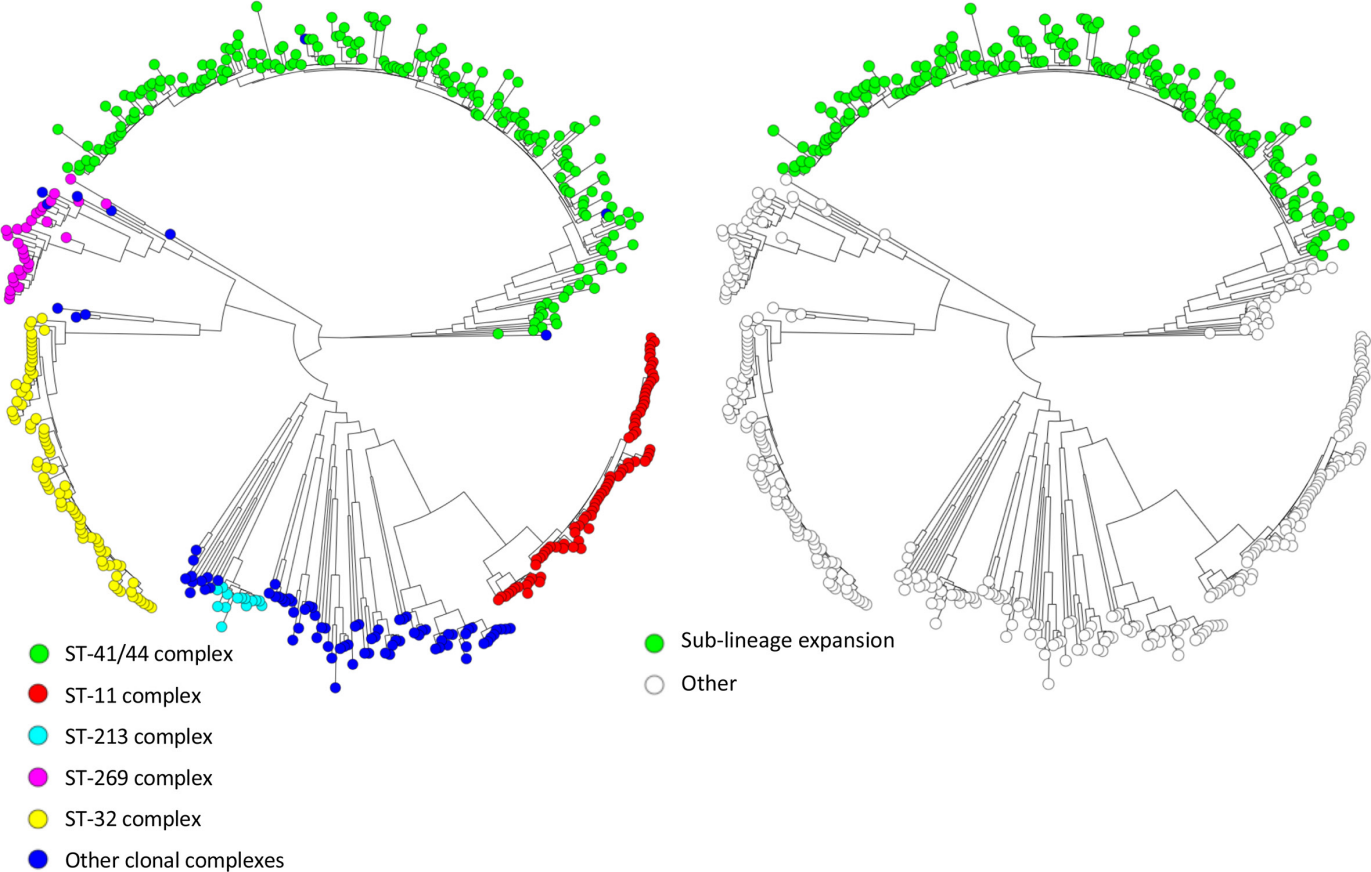
Phylogenetic tree of 486 *Neisseria meningitidis* sequences showing major clonal complexes on the left, and the sub-lineage in ST-41/44 complex on the right.

Bacterial whole-genome sequencing was performed on 489 isolates. After quality control, three isolates were excluded (contamination in 1, sequencing and assembly issues in 2), leaving 486 sequences for analysis. Of these, 354 isolates (73 %) were serogroup B and 107 isolates (22 %) serogroup C. The remaining 25 isolates (5 %) were serogroup A, W, X, Y or E. Genetic analysis showed 154 unique sequence types (STs), with most sequences belonging to three large clonal complex groups. Of 486 isolates, 197 (41 %) were ST-41/44 complex (98% serogroup B), 81 (17 %) were ST-11 complex (99% serogroup C) and 80 (16%) were ST-32 complex (100% serogroup B). In 1979–2003 the proportion of ST-41/44 complex isolates was higher than that in 2006–2013 [134 of 290 (46 %) versus 63 of 196 (32 %), chi-square *P*=0.002, Fig. 1]. The proportion of ST-32 complex isolates was higher in the later time period [40 of 290 (14 %), versus 40 of 196 (29 %), chi-square *P*=0.054, Fig. 1]. To further investigate the evolutionary relationships between isolates, a phylogenetic tree was generated from the core-genome sequences. The phylogenetic tree has long branches between ancestral nodes and between nodes and tips, a feature consistent with genetic variability in this species (Fig. 2). Closer examination by a Bayesian clustering analysis (hierBAPS) reveals a sub-lineage within the ST-41/44 complex clade, comprising 178/197 (90 %) ST-41/44 isolates (Figs 2 and S2). By correlating genetic distance to sampling date in a Bayesian evolutionary analysis of trees (BEAST), it was determined that the most recent common ancestor to isolates in the ST-41/44 sub-lineage arose 56 years (95% confidence interval 47–74 years) ago (Fig. S3). The most recent common ancestor to ST-32 complex emerged 147 years (95% confidence interval 98–241 years) ago (Fig. S4). This suggests the ST-41/44 sub-lineage emerged fairly recently compared to the other major serogroup B clade. Taken together, ST-41/44 sub-lineage was the main determinant of serogroup B prevalence during the hyperendemic period. We next sought to determine the distribution of antigen allele variants over clades in the phylogeny.

### Dominant antigen alleles varied over the study period

**Fig. 3.**
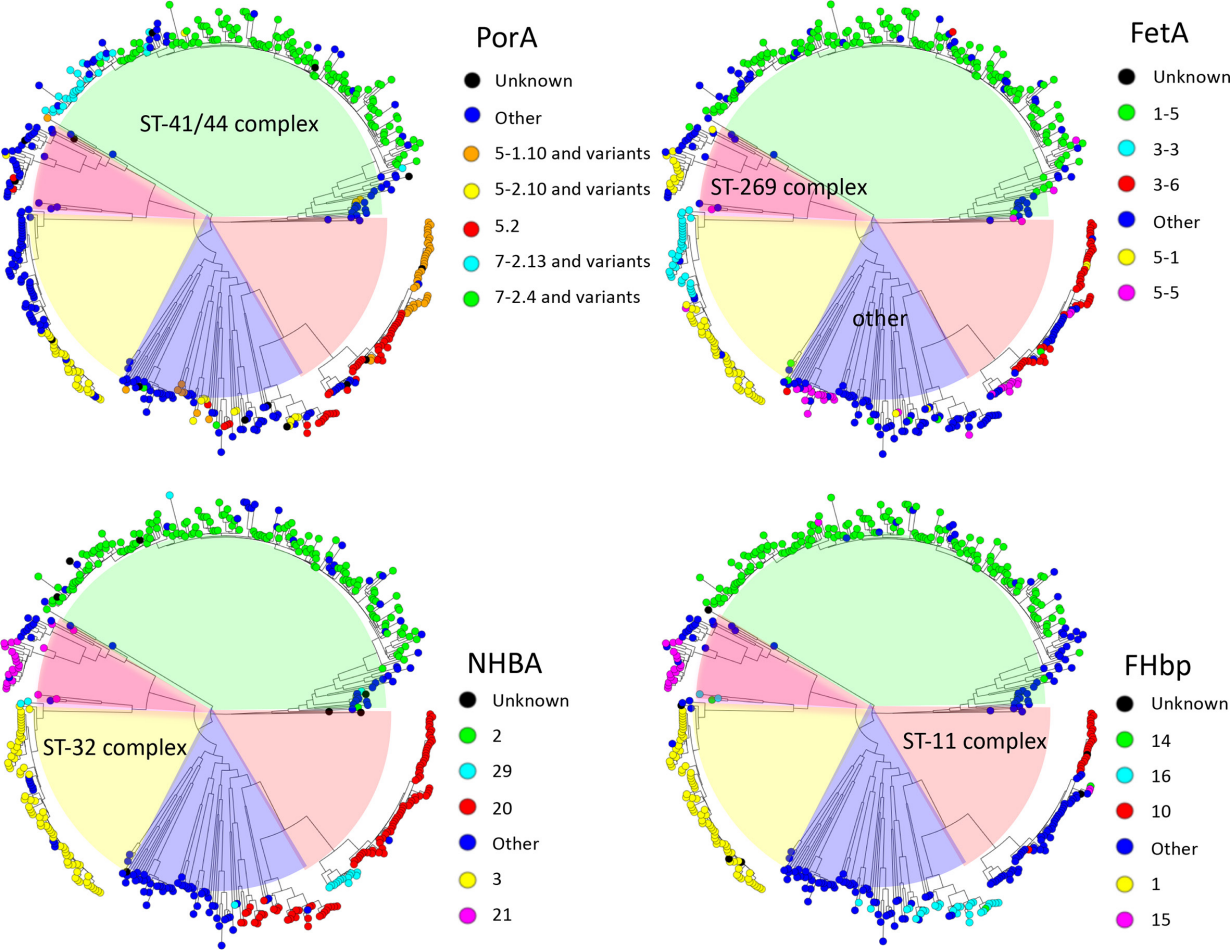
Antigen alleles over the phylogenetic tree cluster with clonal complexes. The upper left shows major PorA alleles, upper right major FetA alleles, lower left major NHBA alleles and lower right major FHbp alleles over the phylogenetic tree.

**Fig. 4.**
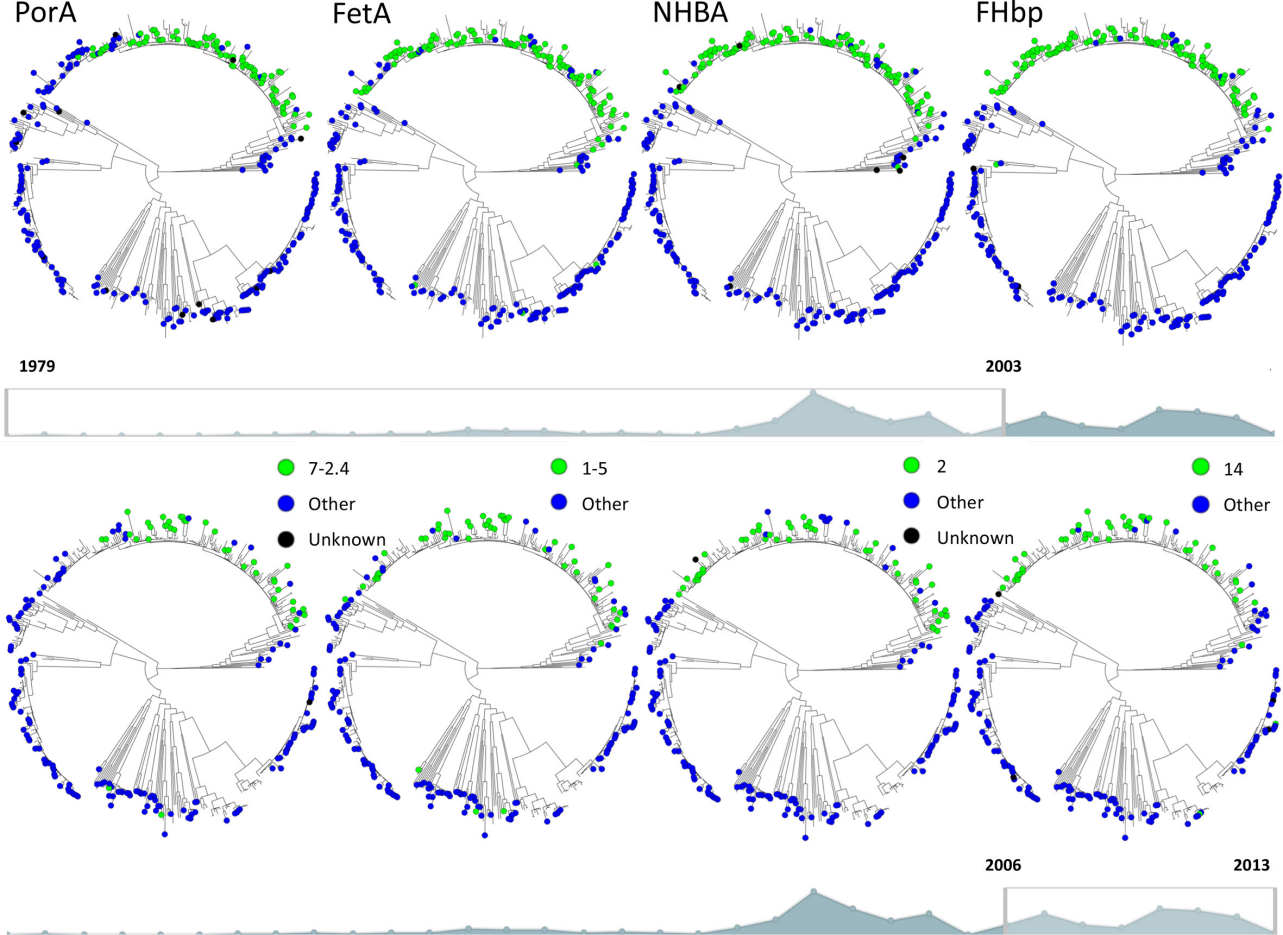
PorA, FetA, NHBA and FHbp major peptide alleles over the phylogenetic tree for time periods 1979–2003 (above) and 2006–2013 (below). The major peptide alleles are shown in green and the other alleles in blue. Major peptide alleles cluster with sequences from the ST-41/44 sub-lineage and are more densely present in isolates sampled from 1979 to 2003 (above) compared to 2006–2013 (below).

Antigen peptide allele distribution was assessed for *nhba* and *fHbp* genes and variable regions of *porA* (VR1 and VR2) and *fetA* genes. Among the 486 isolates, 79 PorA, 39 FetA*,* 47 NHBA and 74 FHbp unique peptide alleles were identified. The three most abundant alleles of each antigen comprised 207 of 486 (43 %) for PorA, 267 of 486 (55 %) for FetA, 321 of 486 (66 %) for NHBA and 260 of 486 isolates (53 %) for FHbp. The peptide allele distribution of the assessed antigens showed a non-overlapping distribution among the clonal complexes (Fig. 3). The antigen peptide alleles PorA 7–2.4, FetA 1–5, NHBA two and FHbp 14 are the major alleles among the isolates in the ST-41/44 sub-lineage. Among the 178 isolates in this sub-lineage, 121 (68 %) had PorA 7–2.4, 146 (82 %) had FetA 1–5, 149 (84 %) had NHBA 2 and 153 (86 %) had FHbp 14. Among 308 isolates outside the ST-41/44 sub-lineage PorA 7–2.4 was present in 2 (1 %), FetA 1–5 in 12 (4 %), NHBA 2 in 3 (1 %) and FHbp 14 in 4 (1 %) isolates (Table S3). The major peptide allele distribution among isolates in 1979–2003 differed significantly from that among isolates in 2006–2013; PorA 7–2.4 allele, *P*=0.048; FetA 1–5, *P*=0.017; NHBA 2, *P*=0.012 and FHbp 14, *P*=0.014 (after correcting for multiple testing; Table 1, Fig. 4).

**Table 1.** *P* values for PorA, FetA, NHBA and FHbp major antigen alleles and the association with 1979–2003 versus 2006–2013 time period (Wald test)

Antigen	*P* value	Bonferroni corrected *P* value
PorA		
7–2.4	0.012	0.048
FetA		
1–5	0.0043	0.0172
NHBA		
2	0.0031	0.0124
FHbp		
14	0.0035	0.0140

### Clades show changes in mutation and recombination rates, but similar selection pressure on antigen sequences

The average recombination to mutation ratio was higher for sequences in the ST-41/44 sub-lineage (22.4, 95% confidence interval 19.6–25.2) compared to sequences in the ST-32 clade (8.6, 95% confidence interval 5.9–11.3). Using recombination-corrected phylogenies and analysis of molecular clock rate, we inferred the mutational rate for each of the identified clades. For sequences in the ST-41/44 sub-lineage, the median mutational rate was 4.28 (range 3.59–5.27) nucleotide substitutions per year. For the clade comprising ST-32 complex isolates, the median mutational rate was 1.81 (range 1.28–2.66) nucleotide substitutions per year (Mann–Whitney U test, *P* =<0.001). For antigen genes, we calculated the ratio of non-synonymous to synonymous mutations (d*N*/d*S*). In serogroup B isolates, the ratio for *porA* was 0.65, *fetA* 0.33, *nhba* 0.59 and *fHbp* 0.45. These ratios were comparable for isolates in the ST-41/44 sub-lineage and ST-32 complex or isolates from the later time period (Table S4).

### Vaccine coverage is higher in isolates from the ST-41/44 sub-lineage

**Fig. 5.**
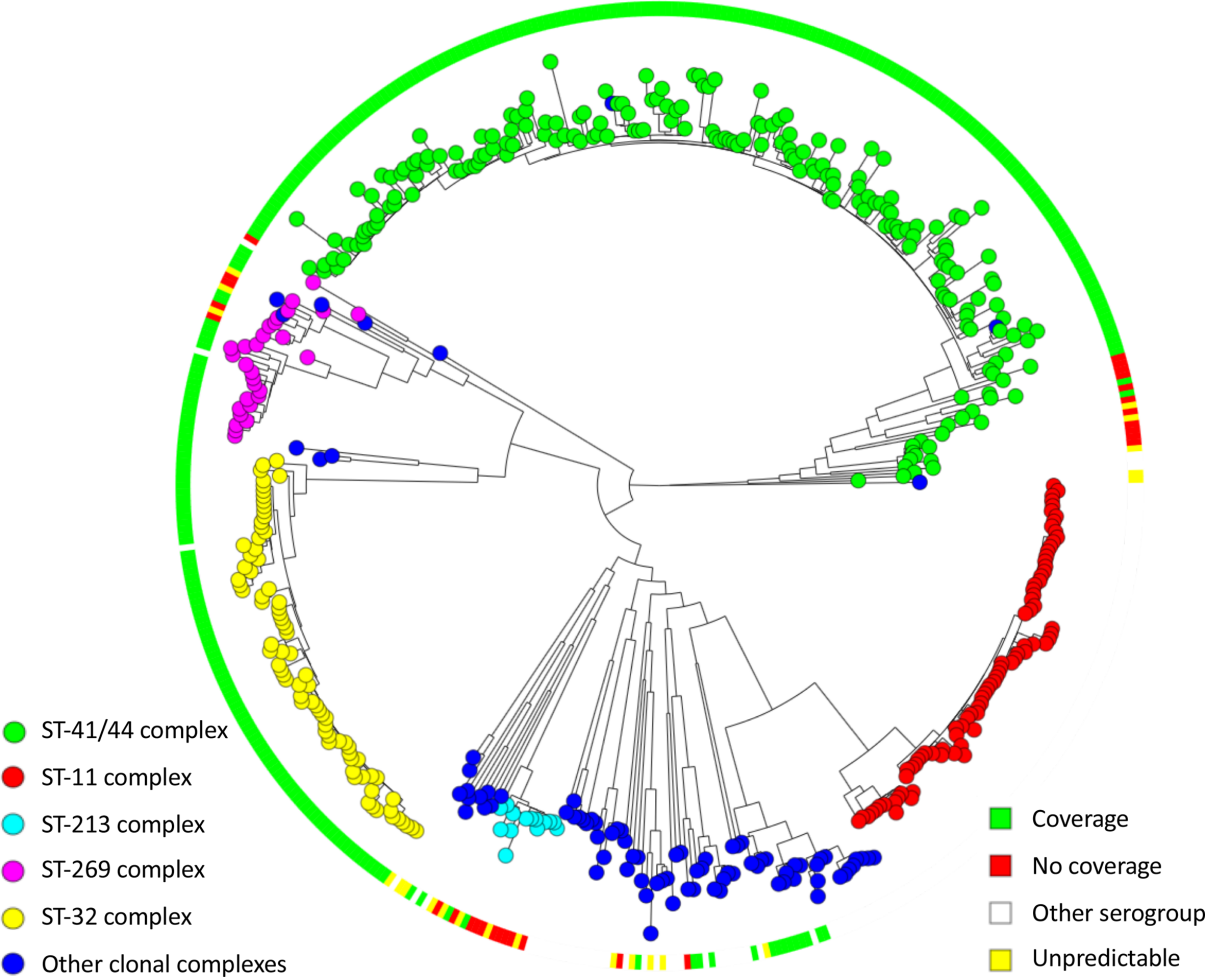
Predicted vaccine coverage for serogroup B isolates. Isolates from ST-32 complex (yellow), the sub-lineage within ST-41/44 complex (green) and other clonal complexes (blue) have 100% predicted vaccine coverage (green marking in the surrounding ring). Isolates from ST-269 complex (magenta), ST-213 (cyan) and ST-41/44 excluding the sub-lineage (green) have predicted vaccine coverage to variable degrees (red and yellow marking in the surrounding ring).

4CMenB vaccine coverage of all serogroup B isolates was assessed by gMATS [27]. Of 354 serogroup B isolates, 305 (86 %) were covered, 28 (8 %) were not covered and for 21 (6 %) isolates the vaccine coverage was unpredictable. For 60 of 305 (20 %) isolates, coverage was based on one peptide. This was FHbp in 29 out of 60 (48 %) and NHBA in 27 out of 60 (45 %) isolates. Isolates in the ST-41/44 sub-lineage had predicted coverage for the 4CMenB vaccine of 100% (178/178 isolates, while all other serogroup B isolates had a predicted coverage of 72 % (127/176); Fisher’s exact test, *P* <0.001). Isolates which were not covered by the vaccine, or for which vaccine coverage was unpredictable, clustered within the phylogeny of the serogroup B population (Fig. 5). The three clonal complexes containing the majority (32/49, 65 %) of these isolates were ST-269 (5/27; 19% not covered or unpredictable coverage), ST-213 (12/14; 86% not covered or unpredictable coverage) and ST-41/44 excluding the sub-lineage (15/18; 83% not covered or unpredictable coverage). Isolates in these clonal complexes (ST-269, ST-213 and ST-41/44 excluding the sub-lineage) showed a trend towards isolation in the latter time period (Fisher’s exact test, *P*=0.08). However, among all serogroup B isolates vaccine coverage was not correlated to time period of isolation (Fisher’s exact test *P*=0.11).

## Discussion

We describe emergence of a sub-lineage within ST-41/44 complex isolates, which subsequently decreased over time and characterized the hyperendemic period in meningococcal meningitis incidence previously described in the Netherlands [2]. This sub-lineage was marked by unique FetA, PorA, NHBA and FHbp peptide allele combinations, of which the last three are the targets of the 4CMenB vaccine. A decrease in the population size of this lineage over time was correlated to a relative decrease in predicted 4CMenB vaccine coverage, illustrating the continuously changing meningococcal outer membrane proteins and associated vaccine coverage. Nevertheless, predicted vaccine coverage for all serogroup B sequences remained high after the hyperendemic period (83 %). The predicted coverage after the hyperendemic period is in line with previous studies reporting 4CMenB coverage rates ranging from 63–90% [28, 29]. Our data illustrate that observed population dynamics of serogroup B meningococcus appears to be driven by changes in size of dominant clonal complexes rather than specific antigen variation independent of this genetic background, although our observational study precludes firm conclusions.

The presence of specific clusters of antigen variants associated with clonal complexes has been previously described as a feature of the meningococcal population [30]. While immune selection acts on individual antigen variants, this study and previous studies show that combinations of antigen variants, which mark discrete lineages persist on a population level [3, 6, 7]. However, when sampling over longer periods of time, as was done in this study, emergence and decline of lineages can be observed, concurrent with changes in dominant antigen variants. This has implications for vaccine development.

Our results do not allow us to draw firm conclusions on whether the evolution of invasive meningococci is driven by changes in size of dominant sequence clusters or specific antigen variants independent of genetic background. We observe non-random and non-overlapping [30] clusters of antigen allele variants associated with clonal complexes, and not intermediate forms of evolving antigen allele variants. Selection pressures by the immune system, fitness of the meningococcal genotype and elimination of variants by bottleneck’s or competition determine the observed population dynamics of sampled invasive isolates [31]. Whole-genome sequencing can further elucidate these intricate relationships.

We found the mutational rate in isolates of the sub-lineage in ST-41/44 complex was higher than in isolates belonging to the ST-32 complex. Mutational rate is dependent on various characteristics of the population under study and this can account for a large variance in the result [32]. Previously published rates in carriage isolates are of the same order of magnitude to what we find in our study. Carriage isolates from the University of Nottingham had a mutation rate of 12.2 nucleotide substitutions per year [33]. In serogroup A carriage isolates from Africa, the mutation rate was 6.2 nucleotide substitutions per year [34]. And in a small cohort of meningococcal carriage isolates, the mutation rate was 1.9 nucleotide substitutions per year [35]. Unpublished results from a large cohort of over 2500 carriage isolates from West Africa, showed the median mutation rate to be 5.94 (range 1.12–11.3). The recombination to mutation ratio in the ST-41/44 sub-lineage was also higher than that of ST-32 complex. Previously published recombination to mutation ratios for *N. meningitidis* are comparable to what is found for ST-32 complex [36, 37]. However, in sequences of clonally related isolates recombination to mutation ratios are in line with what is found for the ST-41/44 sub-lineage [38]. For antigen genes specifically, we found d*N*/d*S* levels indicative of purifying selection, as can be expected for genes under selective pressure by the host immune system. We could not detect subgroups of isolates with antigen genes showing evidence for accumulating escape variants, as might be expected in a declining population such as the sequences from the ST-41/44 complex sub-lineage.

In meningococci, mechanisms of bacterial genetic variation such as phase variation and homologous recombination are well-established sources of antigen structural variability [39]. Among others, *porA* loci have been shown to be hypervariable [40]. In this study hypervariable regions (containing an above average number of mutations) were removed by correcting for recombination. There were no outlier values for mutation rates of the individual sequences in ST-41/44 complex and ST-32 complex.

Our sample set requires a set of caveats on these results. First, we only included *N. meningitidis* causing CSF culture proven meningitis. Though we only used isolates from meningitis, the results are likely to be generalizable to other forms of invasive meningococcal disease, as it has been shown previously that there are no structural genetic differences between sequences isolated from blood and cerebrospinal fluid [16]. Second we included only adult cases. Children have highest disease rate per age group and clinical features and presentation vary among age groups [41, 42]. Third, we were unable to verify results in relation to changes in population dynamics in a matched-population dataset of carriage isolates. Though sequences of meningococcal isolates from carriage in other settings are available, it is unknown to what extent disease causing isolates reflect carriage isolates in our study population [43]. Fourth, we evaluated two restricted time periods (1979–2003 and 2006–2013). Fifth, data on human antibody production against serogroups, and bacterial antigens specifically would have enabled us to further elucidate the host–pathogen interaction preceding, during and following the hyperendemic period.

Whole-genome sequencing allowed us to dissect the contributions of both genetic background and vaccine targeted allele sequences to changing population dynamics. The results demonstrate the importance of monitoring antigen expression from invasive meningococcal isolates over time, and its impact on vaccine development.

## Supplementary Data

Supplementary material 1
