# Supplementary material for: Diversification in immunogenicity genes caused by selective pressures in invasive meningococci
Source: Microb Genom. 2020 Aug 10;6(9):mgen000422. doi: 10.1099/mgen.0.000422 (PMC7643973; doi:10.1099/mgen.0.000422)

Supplementary Table S1.

Sample Name	Accession	Lane Name	Lane Acc	Public Name	Study ID	Study Accession
2842STDY5881	ERS514568	14324_4#1	ERR708610	2071283	2842	ERP004245
2842STDY5881	ERS514569	14324_4#2	ERR708611	2071328	2842	ERP004245
2842STDY5881	ERS514570	14324_4#3	ERR708612	2071344	2842	ERP004245
2842STDY5881	ERS514571	14324_4#4	ERR708613	2071345	2842	ERP004245
2842STDY5881	ERS514572	14324_4#5	ERR708614	2071354	2842	ERP004245
2842STDY5881	ERS514573	14324_4#6	ERR708615	2071442	2842	ERP004245
2842STDY5881	ERS514574	14324_4#7	ERR708616	2071538	2842	ERP004245
2842STDY5881	ERS514575	14324_4#8	ERR708617	2071749	2842	ERP004245
2842STDY5881	ERS514576	14324_4#9	ERR708618	2071759	2842	ERP004245
2842STDY5881	ERS514577	14324_4#10	ERR708619	2071794	2842	ERP004245
2842STDY5881	ERS514578	14324_4#11	ERR708620	2071864	2842	ERP004245
2842STDY5881	ERS514579	14324_4#12	ERR708621	2071909	2842	ERP004245
2842STDY5881	ERS514580	14324_4#13	ERR708622	2080098	2842	ERP004245
2842STDY5881	ERS514581	14324_4#14	ERR708623	2080184	2842	ERP004245
2842STDY5881	ERS514582	14324_4#15	ERR708624	2080418	2842	ERP004245
2842STDY5881	ERS514583	14324_4#16	ERR708625	2080520	2842	ERP004245
2842STDY5881	ERS514584	14324_4#17	ERR708626	2080543	2842	ERP004245
2842STDY5881	ERS514585	14324_4#18	ERR708627	2080556	2842	ERP004245
2842STDY5881	ERS514586	14324_4#19	ERR708628	2080584	2842	ERP004245
2842STDY5881	ERS514587	14324_4#20	ERR708629	2080856	2842	ERP004245
2842STDY5881	ERS514588	14324_4#21	ERR708630	2081063	2842	ERP004245
2842STDY5881	ERS514589	14324_4#22	ERR708631	2081105	2842	ERP004245
2842STDY5881	ERS514590	14324_4#23	ERR708632	2081595	2842	ERP004245
2842STDY5881	ERS514591	14324_4#24	ERR708633	2081653	2842	ERP004245
2842STDY5881	ERS514592	14324_4#25	ERR708634	2081656	2842	ERP004245
2842STDY5881	ERS514593	14324_4#26	ERR708635	2081977	2842	ERP004245
2842STDY5881	ERS514594	14324_4#27	ERR708636	2082123	2842	ERP004245
2842STDY5881	ERS514595	14324_4#28	ERR708637	2082183	2842	ERP004245
2842STDY5881	ERS514596	14324_4#29	ERR708638	2082258	2842	ERP004245
2842STDY5881	ERS514597	14324_4#30	ERR708639	2090237	2842	ERP004245
2842STDY5881	ERS514598	14324_4#31	ERR708640	2090353	2842	ERP004245
2842STDY5881	ERS514599	14324_4#32	ERR708641	2090516	2842	ERP004245
2842STDY5881	ERS514600	14324_4#33	ERR708642	2090911	2842	ERP004245
2842STDY5881	ERS514601	14324_4#34	ERR708643	2091136	2842	ERP004245
2842STDY5881	ERS514602	14324_4#35	ERR708644	2091153	2842	ERP004245
2842STDY5881	ERS514603	14324_4#36	ERR708645	2091198	2842	ERP004245
2842STDY5881	ERS514604	14324_4#37	ERR708646	2091661	2842	ERP004245
2842STDY5881	ERS514605	14324_4#38	ERR708647	2091724	2842	ERP004245
2842STDY5881	ERS514606	14324_4#39	ERR708648	2092127	2842	ERP004245
2842STDY5881	ERS514607	14324_4#40	ERR708649	2093373	2842	ERP004245
2842STDY5881	ERS514608	14324_4#41	ERR708650	2094485	2842	ERP004245
2842STDY5881	ERS514609	14324_4#42	ERR708651	2100125	2842	ERP004245
2842STDY5881	ERS514610	14324_4#43	ERR708652	2100157	2842	ERP004245
2842STDY5881	ERS514611	14324_4#44	ERR708653	2100282	2842	ERP004245

2842STDY5881	ERS514612	14324_4#45	ERR708654	2100381	2842	ERP004245
2842STDY5881	ERS514613	14324_4#46	ERR708655	2100528	2842	ERP004245
2842STDY5881	ERS514614	14324_4#47	ERR708656	2102065	2842	ERP004245
2842STDY5881	ERS514615	14324_4#48	ERR708657	2102083	2842	ERP004245
2842STDY5881	ERS514616	14324_4#49	ERR708658	2103113	2842	ERP004245
2842STDY5881	ERS514617	14324_4#50	ERR708659	2103119	2842	ERP004245
2842STDY5881	ERS514619	14324_4#51	ERR708660	2103251	2842	ERP004245
2842STDY5881	ERS514621	14324_4#52	ERR708661	2103437	2842	ERP004245
2842STDY5881	ERS514623	14324_4#53	ERR708662	2103596	2842	ERP004245
2842STDY5881	ERS514625	14324_4#54	ERR708663	2103742	2842	ERP004245
2842STDY5881	ERS514627	14324_4#55	ERR708664	2103747	2842	ERP004245
2842STDY5881	ERS514628	14324_4#56	ERR708665	2104325	2842	ERP004245
2842STDY5881	ERS514631	14324_4#57	ERR708666	2104366	2842	ERP004245
2842STDY5881	ERS514632	14324_4#58	ERR708667	2110092	2842	ERP004245
2842STDY5881	ERS514634	14324_4#59	ERR708668	2110188	2842	ERP004245
2842STDY5881	ERS514637	14324_4#60	ERR708669	2110538	2842	ERP004245
2842STDY5881	ERS514640	14324_4#61	ERR708670	2110673	2842	ERP004245
2842STDY5881	ERS514642	14324_4#62	ERR708671	2110842	2842	ERP004245
2842STDY5881	ERS514645	14324_4#63	ERR708672	2110915	2842	ERP004245
2842STDY5881	ERS514648	14324_4#64	ERR708673	2110916	2842	ERP004245
2842STDY5881	ERS514651	14324_4#65	ERR708674	2110947	2842	ERP004245
2842STDY5881	ERS514653	14324_4#66	ERR708675	2111171	2842	ERP004245
2842STDY5881	ERS514655	14324_4#67	ERR708676	2111581	2842	ERP004245
2842STDY5881	ERS514658	14324_4#68	ERR708677	2111622	2842	ERP004245
2842STDY5881	ERS514661	14324_4#69	ERR708678	2120056	2842	ERP004245
2842STDY5881	ERS514664	14324_4#70	ERR708679	2120194	2842	ERP004245
2842STDY5881	ERS514667	14324_4#71	ERR708680	2120265	2842	ERP004245
2842STDY5881	ERS514669	14324_4#72	ERR708681	2120394	2842	ERP004245
2842STDY5881	ERS514671	14324_4#73	ERR708682	2120484	2842	ERP004245
2842STDY5881	ERS514674	14324_4#74	ERR708683	2120492	2842	ERP004245
2842STDY5881	ERS514677	14324_4#75	ERR708684	2120610	2842	ERP004245
2842STDY5881	ERS514679	14324_4#76	ERR708685	2120785	2842	ERP004245
2842STDY5881	ERS514681	14324_4#77	ERR708686	2120864	2842	ERP004245
2842STDY5881	ERS514684	14324_4#78	ERR708687	2120910	2842	ERP004245
2842STDY5881	ERS514686	14324_4#79	ERR708688	2120945	2842	ERP004245
2842STDY5881	ERS514689	14324_4#80	ERR708689	2121404	2842	ERP004245
2842STDY5881	ERS514692	14324_4#81	ERR708690	2121460	2842	ERP004245
2842STDY5881	ERS514694	14324_4#82	ERR708691	2121477	2842	ERP004245
2842STDY5881	ERS514696	14324_4#83	ERR708692	2121528	2842	ERP004245
2842STDY5881	ERS514699	14324_4#84	ERR708693	2130047	2842	ERP004245
2842STDY5881	ERS514702	14324_4#85	ERR708694	2130367	2842	ERP004245
2842STDY5881	ERS514705	14324_4#86	ERR708695	2130695	2842	ERP004245
2842STDY5881	ERS514707	14324_4#87	ERR708696	2130749	2842	ERP004245
2842STDY5881	ERS514853	14324_5#1	ERR708705	851200 I	2842	ERP004245
2842STDY5881	ERS514855	14324_5#3	ERR708707	860054 I	2842	ERP004245
2842STDY5881	ERS514857	14324_5#5	ERR708709	860394 I	2842	ERP004245

2842STDY58815	ERS514859	14324_5#7	ERR708711	860524 I		2842	ERP004245
2842STDY58815	ERS514861	14324_5#9	ERR708713	861538 I		2842	ERP004245
2842STDY58815	ERS514863	14324_5#11	ERR708715	871080 I		2842	ERP004245
2842STDY58815	ERS514865	14324_5#13	ERR708717	871091 I		2842	ERP004245
2842STDY58815	ERS514867	14324_5#15	ERR708719	871912 I		2842	ERP004245
2842STDY58815	ERS514869	14324_5#17	ERR708721	880416 I		2842	ERP004245
2842STDY58815	ERS514872	14324_5#20	ERR708724	880417 I		2842	ERP004245
2842STDY58815	ERS514874	14324_5#22	ERR708726	880470 I		2842	ERP004245
2842STDY58815	ERS514876	14324_5#24	ERR708728	881965 I		2842	ERP004245
2842STDY58815	ERS514879	14324_5#27	ERR708731	882011 I		2842	ERP004245
2842STDY58815	ERS514881	14324_5#29	ERR708733	890042 I		2842	ERP004245
2842STDY58815	ERS514884	14324_5#32	ERR708736	890104 I		2842	ERP004245
2842STDY58815	ERS514886	14324_5#34	ERR708738	890681 I		2842	ERP004245
2842STDY58815	ERS514888	14324_5#36	ERR708740	892385 I		2842	ERP004245
2842STDY58815	ERS514890	14324_5#38	ERR708742	892559 I		2842	ERP004245
2842STDY58815	ERS514893	14324_5#41	ERR708745	900382 I		2842	ERP004245
2842STDY58815	ERS514896	14324_5#44	ERR708748	900761 I		2842	ERP004245
2842STDY58815	ERS514898	14324_5#46	ERR708750	900798 I		2842	ERP004245
2842STDY58815	ERS514900	14324_5#48	ERR708752	900932 I		2842	ERP004245
2842STDY58815	ERS514903	14324_5#51	ERR708755	901147 I		2842	ERP004245
2842STDY58815	ERS514906	14324_5#54	ERR708758	901168 I		2842	ERP004245
2842STDY58815	ERS514909	14324_5#57	ERR708761	901458 I		2842	ERP004245
2842STDY58815	ERS514912	14324_5#60	ERR708764	901523 I		2842	ERP004245
2842STDY58815	ERS514914	14324_5#62	ERR708766	901549 I		2842	ERP004245
2842STDY58815	ERS514916	14324_5#64	ERR708768	901833 I		2842	ERP004245
2842STDY58816	ERS514919	14324_5#67	ERR708771	902499 I		2842	ERP004245
2842STDY58816	ERS514922	14324_5#70	ERR708774	910039 I		2842	ERP004245
2842STDY58816	ERS514924	14324_5#72	ERR708776	910083 I		2842	ERP004245
2842STDY58816	ERS514926	14324_5#74	ERR708778	910221 I		2842	ERP004245
2842STDY58816	ERS514928	14324_5#76	ERR708780	910333 I		2842	ERP004245
2842STDY58816	ERS514931	14324_5#79	ERR708783	910763 I		2842	ERP004245
2842STDY58816	ERS514933	14324_5#81	ERR708785	911634 I		2842	ERP004245
2842STDY58816	ERS514935	14324_5#83	ERR708787	911665 I		2842	ERP004245
2842STDY58816	ERS514937	14324_5#85	ERR708789	911685 I		2842	ERP004245
2842STDY58816	ERS514939	14324_5#87	ERR708791	911687 I		2842	ERP004245
2842STDY58816	ERS514942	14324_5#90	ERR708794	920056 I		2842	ERP004245
2842STDY58816	ERS514944	14324_5#92	ERR708796	920293 I		2842	ERP004245
2842STDY58816	ERS514947	14324_5#95	ERR708799	920545 I		2842	ERP004245
2842STDY58816	ERS514284	14355_5#1	ERR715801	2000250		2842	ERP004245
2842STDY58816	ERS514287	14355_5#2	ERR715802	2000297		2842	ERP004245
2842STDY58816	ERS514290	14355_5#3	ERR715803	2000311		2842	ERP004245
2842STDY58816	ERS514294	14355_5#4	ERR715804	2000345		2842	ERP004245
2842STDY58816	ERS514297	14355_5#5	ERR715805	2000373		2842	ERP004245
2842STDY58816	ERS514300	14355_5#6	ERR715806	2000384		2842	ERP004245
2842STDY58816	ERS514304	14355_5#7	ERR715807	2000488		2842	ERP004245
2842STDY58816	ERS514307	14355_5#8	ERR715808	2000500		2842	ERP004245

2842STDY5881(	ERS514310	14355_5#9	ERR715809	2000589	2842	ERP004245
2842STDY5881(	ERS514313	14355_5#10	ERR715810	2000594	2842	ERP004245
2842STDY5881(	ERS514316	14355_5#11	ERR715811	2000607	2842	ERP004245
2842STDY5881(	ERS514318	14355_5#12	ERR715812	2000608	2842	ERP004245
2842STDY5881(	ERS514321	14355_5#13	ERR715813	2000622	2842	ERP004245
2842STDY5881(	ERS514325	14355_5#14	ERR715814	2000709	2842	ERP004245
2842STDY5881(	ERS514328	14355_5#15	ERR715815	2000732	2842	ERP004245
2842STDY5881(	ERS514331	14355_5#16	ERR715816	2000739	2842	ERP004245
2842STDY5881(	ERS514336	14355_5#17	ERR715817	2000749	2842	ERP004245
2842STDY5881(	ERS514338	14355_5#18	ERR715818	2000760	2842	ERP004245
2842STDY5881(	ERS514341	14355_5#19	ERR715819	2000803	2842	ERP004245
2842STDY5881(	ERS514345	14355_5#20	ERR715820	2000804	2842	ERP004245
2842STDY5881(	ERS514348	14355_5#21	ERR715821	2000869	2842	ERP004245
2842STDY5881(	ERS514351	14355_5#22	ERR715822	2000880	2842	ERP004245
2842STDY5881(	ERS514354	14355_5#23	ERR715823	2000881	2842	ERP004245
2842STDY5881(	ERS514357	14355_5#24	ERR715824	2000885	2842	ERP004245
2842STDY5881(	ERS514360	14355_5#25	ERR715825	2000948	2842	ERP004245
2842STDY5881(	ERS514364	14355_5#26	ERR715826	2000974	2842	ERP004245
2842STDY5881(	ERS514367	14355_5#27	ERR715827	2000987	2842	ERP004245
2842STDY5881(	ERS514371	14355_5#28	ERR715828	2001043	2842	ERP004245
2842STDY5881(	ERS514374	14355_5#29	ERR715829	2001212	2842	ERP004245
2842STDY5881(	ERS514377	14355_5#30	ERR715830	2001256	2842	ERP004245
2842STDY5881(	ERS514381	14355_5#31	ERR715831	2001297	2842	ERP004245
2842STDY5881(	ERS514384	14355_5#32	ERR715832	2001318	2842	ERP004245
2842STDY5881(	ERS514387	14355_5#33	ERR715833	2001329	2842	ERP004245
2842STDY5881(	ERS514391	14355_5#34	ERR715834	2001346	2842	ERP004245
2842STDY5881(	ERS514394	14355_5#35	ERR715835	2001394	2842	ERP004245
2842STDY5881(	ERS514397	14355_5#36	ERR715836	2001449	2842	ERP004245
2842STDY5881(	ERS514400	14355_5#37	ERR715837	2001474	2842	ERP004245
2842STDY5881(	ERS514404	14355_5#38	ERR715838	2001477	2842	ERP004245
2842STDY5881(	ERS514407	14355_5#39	ERR715839	2001553	2842	ERP004245
2842STDY5881(	ERS514410	14355_5#40	ERR715840	2001556	2842	ERP004245
2842STDY5881(	ERS514413	14355_5#41	ERR715841	2001573	2842	ERP004245
2842STDY5881(	ERS514416	14355_5#42	ERR715842	2001615	2842	ERP004245
2842STDY5881(	ERS514419	14355_5#43	ERR715843	2001632	2842	ERP004245
2842STDY5881(	ERS514422	14355_5#44	ERR715844	2001633	2842	ERP004245
2842STDY5881(	ERS514425	14355_5#45	ERR715845	2001717	2842	ERP004245
2842STDY5881(	ERS514429	14355_5#46	ERR715846	2001718	2842	ERP004245
2842STDY58811	ERS514433	14355_5#47	ERR715847	2001782	2842	ERP004245
2842STDY58811	ERS514436	14355_5#48	ERR715848	2001862	2842	ERP004245
2842STDY58811	ERS514440	14355_5#49	ERR715849	2001915	2842	ERP004245
2842STDY58811	ERS514443	14355_5#50	ERR715850	2001970	2842	ERP004245
2842STDY58811	ERS514447	14355_5#51	ERR715851	2002042	2842	ERP004245
2842STDY58811	ERS514452	14355_5#52	ERR715852	2002091	2842	ERP004245
2842STDY58811	ERS514455	14355_5#53	ERR715853	2002154	2842	ERP004245
2842STDY58811	ERS514459	14355_5#54	ERR715854	2002173	2842	ERP004245

2842STDY5881	ERS514461	14355_5#55	ERR715855	2010002	2842	ERP004245
2842STDY5881	ERS514464	14355_5#56	ERR715856	2010024	2842	ERP004245
2842STDY5881	ERS514468	14355_5#57	ERR715857	2010080	2842	ERP004245
2842STDY5881	ERS514472	14355_5#58	ERR715858	2010082	2842	ERP004245
2842STDY5881	ERS514476	14355_5#59	ERR715859	2010115	2842	ERP004245
2842STDY5881	ERS514480	14355_5#60	ERR715860	2010178	2842	ERP004245
2842STDY5881	ERS514483	14355_5#61	ERR715861	2010216	2842	ERP004245
2842STDY5881	ERS514486	14355_5#62	ERR715862	2010221	2842	ERP004245
2842STDY5881	ERS514490	14355_5#63	ERR715863	2010259	2842	ERP004245
2842STDY5881	ERS514493	14355_5#64	ERR715864	2010277	2842	ERP004245
2842STDY5881	ERS514495	14355_5#65	ERR715865	2010306	2842	ERP004245
2842STDY5881	ERS514498	14355_5#66	ERR715866	2010321	2842	ERP004245
2842STDY5881	ERS514503	14355_5#67	ERR715867	2010353	2842	ERP004245
2842STDY5881	ERS514506	14355_5#68	ERR715868	2010450	2842	ERP004245
2842STDY5881	ERS514509	14355_5#69	ERR715869	2010513	2842	ERP004245
2842STDY5881	ERS514512	14355_5#70	ERR715870	2010640	2842	ERP004245
2842STDY5881	ERS514515	14355_5#71	ERR715871	2010688	2842	ERP004245
2842STDY5881	ERS514518	14355_5#72	ERR715872	2010699	2842	ERP004245
2842STDY5881	ERS514522	14355_5#73	ERR715873	2010749	2842	ERP004245
2842STDY5881	ERS514525	14355_5#74	ERR715874	2010760	2842	ERP004245
2842STDY5881	ERS514528	14355_5#75	ERR715875	2010903	2842	ERP004245
2842STDY5881	ERS514530	14355_5#76	ERR715876	2010904	2842	ERP004245
2842STDY5881	ERS514532	14355_5#77	ERR715877	2010939	2842	ERP004245
2842STDY5881	ERS514534	14355_5#78	ERR715878	2010999	2842	ERP004245
2842STDY5881	ERS514536	14355_5#79	ERR715879	2011023	2842	ERP004245
2842STDY5881	ERS514537	14355_5#80	ERR715880	2011029	2842	ERP004245
2842STDY5881	ERS514539	14355_5#81	ERR715881	2011043	2842	ERP004245
2842STDY5881	ERS514542	14355_5#82	ERR715882	2011060	2842	ERP004245
2842STDY5881	ERS514543	14355_5#83	ERR715883	2011098	2842	ERP004245
2842STDY5881	ERS514545	14355_5#84	ERR715884	2011148	2842	ERP004245
2842STDY5881	ERS514547	14355_5#85	ERR715885	2011150	2842	ERP004245
2842STDY5881	ERS514549	14355_5#86	ERR715886	2011212	2842	ERP004245
2842STDY5881	ERS514551	14355_5#87	ERR715887	2011215	2842	ERP004245
2842STDY5881	ERS514553	14355_5#88	ERR715888	2011233	2842	ERP004245
2842STDY5881	ERS514555	14355_5#89	ERR715889	2011246	2842	ERP004245
2842STDY5881	ERS514557	14355_5#90	ERR715890	2011332	2842	ERP004245
2842STDY5881	ERS514559	14355_5#91	ERR715891	2011334	2842	ERP004245
2842STDY5881	ERS514560	14355_5#92	ERR715892	2011337	2842	ERP004245
2842STDY5881	ERS514563	14355_5#93	ERR715893	2011528	2842	ERP004245
2842STDY5881	ERS514565	14355_5#94	ERR715894	2011564	2842	ERP004245
2842STDY5881	ERS514566	14355_5#95	ERR715895	2011595	2842	ERP004245
2842STDY5881	ERS514286	14355_6#1	ERR715896	2011728	2842	ERP004245
2842STDY5881	ERS514289	14355_6#2	ERR715897	2011745	2842	ERP004245
2842STDY5881	ERS514292	14355_6#3	ERR715898	2011764	2842	ERP004245
2842STDY5881	ERS514293	14355_6#4	ERR715899	2011814	2842	ERP004245
2842STDY5881	ERS514296	14355_6#5	ERR715900	2011831	2842	ERP004245

2842STDY5881	ERS514299	14355_6#6	ERR715901	2011832	2842	ERP004245
2842STDY5881	ERS514301	14355_6#7	ERR715902	2011833	2842	ERP004245
2842STDY5881	ERS514303	14355_6#8	ERR715903	2011851	2842	ERP004245
2842STDY5881	ERS514305	14355_6#9	ERR715904	2011973	2842	ERP004245
2842STDY5881	ERS514308	14355_6#10	ERR715905	2011979	2842	ERP004245
2842STDY5881	ERS514311	14355_6#11	ERR715906	2012202	2842	ERP004245
2842STDY5881	ERS514314	14355_6#12	ERR715907	2012239	2842	ERP004245
2842STDY5881	ERS514317	14355_6#13	ERR715908	2012278	2842	ERP004245
2842STDY5881	ERS514319	14355_6#14	ERR715909	2012280	2842	ERP004245
2842STDY5881	ERS514322	14355_6#15	ERR715910	2012303	2842	ERP004245
2842STDY5881	ERS514324	14355_6#16	ERR715911	2012326	2842	ERP004245
2842STDY5881	ERS514327	14355_6#17	ERR715912	2012431	2842	ERP004245
2842STDY5881	ERS514330	14355_6#18	ERR715913	2012552	2842	ERP004245
2842STDY5881	ERS514332	14355_6#19	ERR715914	2012598	2842	ERP004245
2842STDY5881	ERS514334	14355_6#20	ERR715915	2012602	2842	ERP004245
2842STDY5881	ERS514339	14355_6#21	ERR715916	2012620	2842	ERP004245
2842STDY5881	ERS514340	14355_6#22	ERR715917	2012640	2842	ERP004245
2842STDY5881	ERS514343	14355_6#23	ERR715918	2012655	2842	ERP004245
2842STDY5881	ERS514346	14355_6#24	ERR715919	2012673	2842	ERP004245
2842STDY5881	ERS514349	14355_6#25	ERR715920	2020047	2842	ERP004245
2842STDY5881	ERS514355	14355_6#27	ERR715921	2020149	2842	ERP004245
2842STDY5881	ERS514358	14355_6#28	ERR715922	2020150	2842	ERP004245
2842STDY5881	ERS514359	14355_6#29	ERR715923	2020151	2842	ERP004245
2842STDY5881	ERS514362	14355_6#30	ERR715924	2020165	2842	ERP004245
2842STDY5881	ERS514365	14355_6#31	ERR715925	2020193	2842	ERP004245
2842STDY5881	ERS514368	14355_6#32	ERR715926	2020207	2842	ERP004245
2842STDY5881	ERS514370	14355_6#33	ERR715927	2020226	2842	ERP004245
2842STDY5881	ERS514372	14355_6#34	ERR715928	2020276	2842	ERP004245
2842STDY5881	ERS514375	14355_6#35	ERR715929	2020324	2842	ERP004245
2842STDY5881	ERS514378	14355_6#36	ERR715930	2020328	2842	ERP004245
2842STDY5881	ERS514380	14355_6#37	ERR715931	2020383	2842	ERP004245
2842STDY5881	ERS514382	14355_6#38	ERR715932	2020416	2842	ERP004245
2842STDY5881	ERS514385	14355_6#39	ERR715933	2020417	2842	ERP004245
2842STDY5881	ERS514388	14355_6#40	ERR715934	2020434	2842	ERP004245
2842STDY5881	ERS514390	14355_6#41	ERR715935	2020435	2842	ERP004245
2842STDY5881	ERS514392	14355_6#42	ERR715936	2020449	2842	ERP004245
2842STDY5881	ERS514395	14355_6#43	ERR715937	2020473	2842	ERP004245
2842STDY5881	ERS514398	14355_6#44	ERR715938	2020479	2842	ERP004245
2842STDY5881	ERS514401	14355_6#45	ERR715939	2020503	2842	ERP004245
2842STDY5881	ERS514402	14355_6#46	ERR715940	2020546	2842	ERP004245
2842STDY5881	ERS514405	14355_6#47	ERR715941	2020547	2842	ERP004245
2842STDY5881	ERS514408	14355_6#48	ERR715942	2020561	2842	ERP004245
2842STDY5881	ERS514411	14355_6#49	ERR715943	2020622	2842	ERP004245
2842STDY5881	ERS514414	14355_6#50	ERR715944	2020707	2842	ERP004245
2842STDY5881:	ERS514417	14355_6#51	ERR715945	2020745	2842	ERP004245
2842STDY5881:	ERS514421	14355_6#52	ERR715946	2020786	2842	ERP004245

2842STDY5881	ERS514423	14355_6#53	ERR715947	2020798	2842	ERP004245
2842STDY5881	ERS514426	14355_6#54	ERR715948	2020799	2842	ERP004245
2842STDY5881	ERS514427	14355_6#55	ERR715949	2020843	2842	ERP004245
2842STDY5881	ERS514430	14355_6#56	ERR715950	2060092	2842	ERP004245
2842STDY5881	ERS514432	14355_6#57	ERR715951	2060516	2842	ERP004245
2842STDY5881	ERS514434	14355_6#58	ERR715952	2060603	2842	ERP004245
2842STDY5881	ERS514437	14355_6#59	ERR715953	2060640	2842	ERP004245
2842STDY5881	ERS514439	14355_6#60	ERR715954	2060716	2842	ERP004245
2842STDY5881	ERS514442	14355_6#61	ERR715955	2060737	2842	ERP004245
2842STDY5881	ERS514444	14355_6#62	ERR715956	2060979	2842	ERP004245
2842STDY5881	ERS514446	14355_6#63	ERR715957	2061025	2842	ERP004245
2842STDY5881	ERS514449	14355_6#64	ERR715958	2061079	2842	ERP004245
2842STDY5881	ERS514450	14355_6#65	ERR715959	2061250	2842	ERP004245
2842STDY5881	ERS514453	14355_6#66	ERR715960	2061255	2842	ERP004245
2842STDY5881	ERS514457	14355_6#68	ERR715961	2061370	2842	ERP004245
2842STDY5881	ERS514460	14355_6#69	ERR715962	2061400	2842	ERP004245
2842STDY5881	ERS514463	14355_6#70	ERR715963	2061481	2842	ERP004245
2842STDY5881	ERS514465	14355_6#71	ERR715964	2061515	2842	ERP004245
2842STDY5881	ERS514467	14355_6#72	ERR715965	2061596	2842	ERP004245
2842STDY5881	ERS514470	14355_6#73	ERR715966	2061655	2842	ERP004245
2842STDY5881	ERS514473	14355_6#74	ERR715967	2070069	2842	ERP004245
2842STDY5881	ERS514475	14355_6#75	ERR715968	2070080	2842	ERP004245
2842STDY5881	ERS514477	14355_6#76	ERR715969	2070093	2842	ERP004245
2842STDY5881	ERS514479	14355_6#77	ERR715970	2070150	2842	ERP004245
2842STDY5881	ERS514481	14355_6#78	ERR715971	2070151	2842	ERP004245
2842STDY5881	ERS514484	14355_6#79	ERR715972	2070189	2842	ERP004245
2842STDY5881	ERS514487	14355_6#80	ERR715973	2070215	2842	ERP004245
2842STDY5881	ERS514489	14355_6#81	ERR715974	2070308	2842	ERP004245
2842STDY5881	ERS514491	14355_6#82	ERR715975	2070339	2842	ERP004245
2842STDY5881	ERS514494	14355_6#83	ERR715976	2070395	2842	ERP004245
2842STDY5881	ERS514497	14355_6#84	ERR715977	2070406	2842	ERP004245
2842STDY5881	ERS514500	14355_6#85	ERR715978	2070506	2842	ERP004245
2842STDY5881	ERS514502	14355_6#86	ERR715979	2070591	2842	ERP004245
2842STDY5881	ERS514504	14355_6#87	ERR715980	2070737	2842	ERP004245
2842STDY5881	ERS514507	14355_6#88	ERR715981	2070780	2842	ERP004245
2842STDY5881	ERS514510	14355_6#89	ERR715982	2070862	2842	ERP004245
2842STDY5881	ERS514513	14355_6#90	ERR715983	2070929	2842	ERP004245
2842STDY5881	ERS514516	14355_6#91	ERR715984	2071065	2842	ERP004245
2842STDY5881	ERS514519	14355_6#92	ERR715985	2071109	2842	ERP004245
2842STDY5881	ERS514521	14355_6#93	ERR715986	2071236	2842	ERP004245
2842STDY5881	ERS514524	14355_6#94	ERR715987	2071276	2842	ERP004245
2842STDY5881	ERS514527	14355_6#95	ERR715988	2071282	2842	ERP004245
2842STDY5881	ERS514950	14355_7#3	ERR715991	920550 I	2842	ERP004245
2842STDY5881	ERS514955	14355_7#6	ERR715994	920618 I	2842	ERP004245
2842STDY5881	ERS514959	14355_7#8	ERR715996	920619 I	2842	ERP004245
2842STDY5881	ERS514965	14355_7#11	ERR715999	920858 I	2842	ERP004245

2842STDY5881(	ERS514970	14355_7#13	ERR716001	921206 I		2842	ERP004245
2842STDY5881(	ERS514973	14355_7#15	ERR716003	921940 I		2842	ERP004245
2842STDY5881(	ERS514980	14355_7#18	ERR716006	922049 I		2842	ERP004245
2842STDY5881(	ERS514983	14355_7#20	ERR716008	922173 I		2842	ERP004245
2842STDY5881(	ERS514987	14355_7#22	ERR716010	930164 I		2842	ERP004245
2842STDY5881(	ERS514993	14355_7#25	ERR716013	930470 I		2842	ERP004245
2842STDY5881(	ERS515001	14355_7#29	ERR716017	930524 I		2842	ERP004245
2842STDY5881(	ERS515005	14355_7#31	ERR716019	930633 I		2842	ERP004245
2842STDY5881(	ERS515013	14355_7#35	ERR716023	931069 I		2842	ERP004245
2842STDY5881(	ERS515019	14355_7#38	ERR716026	940573 I		2842	ERP004245
2842STDY5881(	ERS515021	14355_7#40	ERR716028	940605 I A		2842	ERP004245
2842STDY5881(	ERS515022	14355_7#41	ERR716029	940605 I B		2842	ERP004245
2842STDY5881(	ERS515024	14355_7#43	ERR716031	940655 I		2842	ERP004245
2842STDY5881(	ERS515026	14355_7#45	ERR716033	940895 I		2842	ERP004245
2842STDY5881(	ERS515028	14355_7#47	ERR716035	940908 I		2842	ERP004245
2842STDY5881(	ERS515031	14355_7#50	ERR716038	941761 I		2842	ERP004245
2842STDY5881(	ERS515034	14355_7#53	ERR716041	950733 I		2842	ERP004245
2842STDY5881(	ERS515037	14355_7#56	ERR716044	951018 I		2842	ERP004245
2842STDY5881(	ERS515039	14355_7#58	ERR716046	951249 I		2842	ERP004245
2842STDY5881(	ERS515042	14355_7#61	ERR716049	951529 I		2842	ERP004245
2842STDY5881(	ERS515044	14355_7#63	ERR716051	960444 I		2842	ERP004245
2842STDY5881(	ERS515047	14355_7#66	ERR716054	961265 I		2842	ERP004245
2842STDY5881(	ERS515049	14355_7#68	ERR716056	962030 I		2842	ERP004245
2842STDY5881(	ERS515051	14355_7#70	ERR716058	970208 I		2842	ERP004245
2842STDY5881'	ERS515053	14355_7#72	ERR716060	970710 I		2842	ERP004245
2842STDY5881'	ERS515055	14355_7#74	ERR716062	971589 I		2842	ERP004245
2842STDY5881'	ERS515058	14355_7#77	ERR716065	971612 I		2842	ERP004245
2842STDY5881'	ERS515060	14355_7#79	ERR716067	971859 I		2842	ERP004245
2842STDY5881'	ERS515062	14355_7#81	ERR716069	971982 I		2842	ERP004245
2842STDY5881'	ERS515064	14355_7#83	ERR716071	980082 I		2842	ERP004245
2842STDY5881'	ERS515066	14355_7#85	ERR716073	980748 I		2842	ERP004245
2842STDY5881'	ERS515069	14355_7#88	ERR716076	980792 I		2842	ERP004245
2842STDY5881'	ERS515072	14355_7#91	ERR716079	981287 I		2842	ERP004245
2842STDY5881'	ERS515075	14355_7#94	ERR716082	981338 I		2842	ERP004245
2842STDY5881'	ERS514954	14355_8#2	ERR716085	981564 I		2842	ERP004245
2842STDY5881'	ERS514960	14355_8#5	ERR716088	982330 I		2842	ERP004245
2842STDY5881'	ERS514966	14355_8#8	ERR716091	990447 I		2842	ERP004245
2842STDY5881'	ERS514972	14355_8#11	ERR716094	990612 I		2842	ERP004245
2842STDY5881'	ERS514977	14355_8#14	ERR716097	991441 I		2842	ERP004245
2842STDY5881'	ERS514981	14355_8#16	ERR716099	991933 I		2842	ERP004245
2842STDY5881'	ERS514988	14355_8#19	ERR716102	2000202 I		2842	ERP004245
2842STDY5881'	ERS514994	14355_8#22	ERR716105	2010815 I		2842	ERP004245
2842STDY5881'	ERS514997	14355_8#24	ERR716107	2010934 I		2842	ERP004245
2842STDY5881'	ERS515002	14355_8#26	ERR716109	2011245 I		2842	ERP004245
2842STDY5881'	ERS515008	14355_8#29	ERR716112	2012554 I		2842	ERP004245
2842STDY5881'	ERS515012	14355_8#31	ERR716114	2030722 I		2842	ERP004245

2842STDY58817	ERS515016	14355_8#33	ERR716116	2031439 I	2842	ERP004245
2842STDY58814	ERS514770	14555_6#51	ERR731263	970636	2842	ERP004245
2842STDY58814	ERS514772	14555_6#52	ERR731264	970701	2842	ERP004245
2842STDY58814	ERS514774	14555_6#53	ERR731265	970974	2842	ERP004245
2842STDY58814	ERS514776	14555_6#54	ERR731266	971285	2842	ERP004245
2842STDY58814	ERS514778	14555_6#55	ERR731267	972054	2842	ERP004245
2842STDY58814	ERS514781	14555_6#57	ERR731269	980025	2842	ERP004245
2842STDY58814	ERS514784	14555_6#58	ERR731270	981146	2842	ERP004245
2842STDY58814	ERS514788	14555_6#60	ERR731272	990540	2842	ERP004245
2842STDY58814	ERS514792	14555_6#62	ERR731274	991076	2842	ERP004245
2842STDY58815	ERS514794	14555_6#63	ERR731275	2001532	2842	ERP004245
2842STDY58815	ERS514796	14555_6#64	ERR731276	780733 I	2842	ERP004245
2842STDY58815	ERS514800	14555_6#66	ERR731278	790143 I	2842	ERP004245
2842STDY58815	ERS514804	14555_6#68	ERR731280	790200 I	2842	ERP004245
2842STDY58815	ERS514810	14555_6#71	ERR731283	790555 I	2842	ERP004245
2842STDY58815	ERS514816	14555_6#74	ERR731286	800879 I	2842	ERP004245
2842STDY58815	ERS514820	14555_6#76	ERR731288	811198 I	2842	ERP004245
2842STDY58815	ERS514826	14555_6#79	ERR731291	820287 I	2842	ERP004245
2842STDY58815	ERS514830	14555_6#81	ERR731293	830088 I	2842	ERP004245
2842STDY58815	ERS514835	14555_6#84	ERR731296	840395 I	2842	ERP004245
2842STDY58815	ERS514839	14555_6#86	ERR731298	841159 I	2842	ERP004245
2842STDY58815	ERS514843	14555_6#88	ERR731300	841367 I	2842	ERP004245
2842STDY58815	ERS514848	14555_6#91	ERR731303	850782 I	2842	ERP004245
2842STDY58815	ERS514850	14555_6#93	ERR731305	850955 I	2842	ERP004245
2842STDY58809	ERS514283	14672_1#1	ERR743244	981781	2842	ERP004245
2842STDY58809	ERS514285	14672_1#2	ERR743245	981784	2842	ERP004245
2842STDY58809	ERS514288	14672_1#3	ERR743246	981826	2842	ERP004245
2842STDY58809	ERS514291	14672_1#4	ERR743247	981827	2842	ERP004245
2842STDY58809	ERS514295	14672_1#5	ERR743248	981871	2842	ERP004245
2842STDY58809	ERS514298	14672_1#6	ERR743249	981915	2842	ERP004245
2842STDY58809	ERS514302	14672_1#7	ERR743250	981936	2842	ERP004245
2842STDY58809	ERS514306	14672_1#8	ERR743251	982029	2842	ERP004245
2842STDY58809	ERS514309	14672_1#9	ERR743252	982102	2842	ERP004245
2842STDY58809	ERS514312	14672_1#10	ERR743253	982144	2842	ERP004245
2842STDY58809	ERS514315	14672_1#11	ERR743254	982153	2842	ERP004245
2842STDY58809	ERS514320	14672_1#12	ERR743255	982199	2842	ERP004245
2842STDY58809	ERS514323	14672_1#13	ERR743256	982200	2842	ERP004245
2842STDY58809	ERS514326	14672_1#14	ERR743257	982245	2842	ERP004245
2842STDY58809	ERS514329	14672_1#15	ERR743258	982340	2842	ERP004245
2842STDY58809	ERS514333	14672_1#16	ERR743259	982347	2842	ERP004245
2842STDY58809	ERS514335	14672_1#17	ERR743260	982375	2842	ERP004245
2842STDY58809	ERS514337	14672_1#18	ERR743261	990001	2842	ERP004245
2842STDY58809	ERS514342	14672_1#19	ERR743262	990005	2842	ERP004245
2842STDY58809	ERS514344	14672_1#20	ERR743263	990030	2842	ERP004245
2842STDY58809	ERS514347	14672_1#21	ERR743264	990056	2842	ERP004245
2842STDY58809	ERS514350	14672_1#22	ERR743265	990062	2842	ERP004245

2842STDY58809	ERS514353	14672_1#23	ERR743266	990069	2842	ERP004245
2842STDY58809	ERS514356	14672_1#24	ERR743267	990082	2842	ERP004245
2842STDY58809	ERS514361	14672_1#25	ERR743268	990092	2842	ERP004245
2842STDY58809	ERS514363	14672_1#26	ERR743269	990104	2842	ERP004245
2842STDY58809	ERS514366	14672_1#27	ERR743270	990121	2842	ERP004245
2842STDY58809	ERS514369	14672_1#28	ERR743271	990134	2842	ERP004245
2842STDY58809	ERS514373	14672_1#29	ERR743272	990135	2842	ERP004245
2842STDY58809	ERS514376	14672_1#30	ERR743273	990149	2842	ERP004245
2842STDY58809	ERS514379	14672_1#31	ERR743274	990163	2842	ERP004245
2842STDY58809	ERS514383	14672_1#32	ERR743275	990328	2842	ERP004245
2842STDY58809	ERS514386	14672_1#33	ERR743276	990334	2842	ERP004245
2842STDY58809	ERS514389	14672_1#34	ERR743277	990492	2842	ERP004245
2842STDY58809	ERS514393	14672_1#35	ERR743278	990502	2842	ERP004245
2842STDY58809	ERS514396	14672_1#36	ERR743279	990576	2842	ERP004245
2842STDY58809	ERS514399	14672_1#37	ERR743280	990601	2842	ERP004245
2842STDY58809	ERS514403	14672_1#38	ERR743281	990602	2842	ERP004245
2842STDY58809	ERS514406	14672_1#39	ERR743282	990615	2842	ERP004245
2842STDY58809	ERS514409	14672_1#40	ERR743283	990653	2842	ERP004245
2842STDY58809	ERS514412	14672_1#41	ERR743284	990738	2842	ERP004245
2842STDY58809	ERS514415	14672_1#42	ERR743285	990797	2842	ERP004245
2842STDY58810	ERS514418	14672_1#43	ERR743286	990808	2842	ERP004245
2842STDY58810	ERS514420	14672_1#44	ERR743287	990815	2842	ERP004245
2842STDY58810	ERS514424	14672_1#45	ERR743288	990907	2842	ERP004245
2842STDY58810	ERS514428	14672_1#46	ERR743289	990947	2842	ERP004245
2842STDY58810	ERS514431	14672_1#47	ERR743290	990975	2842	ERP004245
2842STDY58810	ERS514435	14672_1#48	ERR743291	991027	2842	ERP004245
2842STDY58810	ERS514441	14672_1#50	ERR743292	991056	2842	ERP004245
2842STDY58810	ERS514445	14672_1#51	ERR743293	991093	2842	ERP004245
2842STDY58810	ERS514448	14672_1#52	ERR743294	991097	2842	ERP004245
2842STDY58810	ERS514451	14672_1#53	ERR743295	991117	2842	ERP004245
2842STDY58810	ERS514454	14672_1#54	ERR743296	991159	2842	ERP004245
2842STDY58810	ERS514458	14672_1#55	ERR743297	991174	2842	ERP004245
2842STDY58810	ERS514462	14672_1#56	ERR743298	991192	2842	ERP004245
2842STDY58810	ERS514466	14672_1#57	ERR743299	991208	2842	ERP004245
2842STDY58810	ERS514469	14672_1#58	ERR743300	991210	2842	ERP004245
2842STDY58810	ERS514471	14672_1#59	ERR743301	991247	2842	ERP004245
2842STDY58810	ERS514474	14672_1#60	ERR743302	991275	2842	ERP004245
2842STDY58810	ERS514478	14672_1#61	ERR743303	991344	2842	ERP004245
2842STDY58810	ERS514482	14672_1#62	ERR743304	991379	2842	ERP004245
2842STDY58810	ERS514485	14672_1#63	ERR743305	991382	2842	ERP004245
2842STDY58810	ERS514488	14672_1#64	ERR743306	991397	2842	ERP004245
2842STDY58810	ERS514492	14672_1#65	ERR743307	991511	2842	ERP004245
2842STDY58810	ERS514496	14672_1#66	ERR743308	991598	2842	ERP004245
2842STDY58810	ERS514499	14672_1#67	ERR743309	991625	2842	ERP004245
2842STDY58810	ERS514501	14672_1#68	ERR743310	991642	2842	ERP004245
2842STDY58810	ERS514505	14672_1#69	ERR743311	991661	2842	ERP004245

2842STDY5881[illegible]	ERS514511	14672_1#71	ERR743313	991712	2842	ERP004245
2842STDY5881[illegible]	ERS514514	14672_1#72	ERR743314	991722	2842	ERP004245
2842STDY5881[illegible]	ERS514520	14672_1#74	ERR743315	991774	2842	ERP004245
2842STDY5881[illegible]	ERS514523	14672_1#75	ERR743316	991853	2842	ERP004245
2842STDY5881[illegible]	ERS514526	14672_1#76	ERR743317	991930	2842	ERP004245
2842STDY5881[illegible]	ERS514529	14672_1#77	ERR743318	991932	2842	ERP004245
2842STDY5881[illegible]	ERS514531	14672_1#78	ERR743319	992008	2842	ERP004245
2842STDY5881[illegible]	ERS514533	14672_1#79	ERR743320	992062	2842	ERP004245
2842STDY5881[illegible]	ERS514535	14672_1#80	ERR743321	992073	2842	ERP004245
2842STDY5881[illegible]	ERS514538	14672_1#81	ERR743322	992076	2842	ERP004245
2842STDY5881[illegible]	ERS514540	14672_1#82	ERR743323	2000020	2842	ERP004245
2842STDY5881[illegible]	ERS514541	14672_1#83	ERR743324	2000024	2842	ERP004245
2842STDY5881[illegible]	ERS514544	14672_1#84	ERR743325	2000041	2842	ERP004245
2842STDY5881[illegible]	ERS514546	14672_1#85	ERR743326	2000070	2842	ERP004245
2842STDY5881[illegible]	ERS514548	14672_1#86	ERR743327	2000100	2842	ERP004245
2842STDY5881[illegible]	ERS514550	14672_1#87	ERR743328	2000101	2842	ERP004245
2842STDY5881[illegible]	ERS514552	14672_1#88	ERR743329	2000131	2842	ERP004245
2842STDY5881[illegible]	ERS514554	14672_1#89	ERR743330	2000136	2842	ERP004245
2842STDY5881[illegible]	ERS514556	14672_1#90	ERR743331	2000149	2842	ERP004245
2842STDY5881[illegible]	ERS514558	14672_1#91	ERR743332	2000151	2842	ERP004245
2842STDY5881[illegible]	ERS514561	14672_1#92	ERR743333	2000194	2842	ERP004245
2842STDY5881[illegible]	ERS514562	14672_1#93	ERR743334	2000201	2842	ERP004245
2842STDY5881[illegible]	ERS514564	14672_1#94	ERR743335	2000234	2842	ERP004245
2842STDY5881[illegible]	ERS514567	14672_1#95	ERR743336	2000236	2842	ERP004245
2842STDY5881[illegible]	ERS514438	14893_3#67	ERR775252	991044	2842	ERP004245
2842STDY5881[illegible]	ERS514517	14893_3#68	ERR775253	991765	2842	ERP004245
2842STDY5881[illegible]	ERS514352	14893_3#69	ERR775254	2020094	2842	ERP004245
2842STDY5881[illegible]	ERS514456	14893_3#70	ERR775255	2061285	2842	ERP004245

Supplementary Table S2.

lane ID	serogroup	complex	porA	fetA	fHbp	NHBA	year
14324_4#1	B	ST-32	5-2.10	5-1	1	3	2007
14324_4#10	B	ST-32	5-2.10	5-1	1	3	2007
14324_4#11	B	ST-41/44	22.14-6	1-5	4	2	2007
14324_4#12	B	ST-32	19.13	5-1	1	3	2007
14324_4#13	C	ST-11	5-1.10-8	3-6	15	20	2008
14324_4#14	C	ST-11	5-1.10-8	3-6	14	20	2008
14324_4#15	B	ST-41/44	18.25	1-5	14	2	2008
14324_4#16	B	ST-41/44	7-2.13-2	4-1	14	2	2008
14324_4#17	B	ST-41/44	7-2.4	1-5	14	2	2008
14324_4#18	B	ST-41/44	7-2.4	1-5	31	2	2008
14324_4#19	C	ST-334	18-1.30	3-7	19	6	2008
14324_4#2	B	ST-41/44	7-2.4	1-5	14	2	2007
14324_4#20	B	ST-41/44	7-2.4	1-5	14	2	2008
14324_4#21	B	ST-269	22.9	5-12	4	17	2008
14324_4#22	B	ST-32	7-2.16-99	3-49	1	94	2008
14324_4#23	B	ST-213	22.14	1-7	45	18	2008
14324_4#24	B	ST-41/44	18-1.3	1-5	15	2	2008
14324_4#25	B	ST-32	7.16-32	3-3	1	3	2008
14324_4#26	B	ST-41/44	7-2.4	1-5	14	29	2008
14324_4#27	B	ST-41/44	7-2.13-2	1-5	14	2	2008
14324_4#28	B	ST-32	5-2.10	5-1	1	3	2008
14324_4#29	B	ST-41/44	7-2.4	1-5	14	2	2008
14324_4#3	B	ST-41/44	7-2.4	1-5	13	2	2007
14324_4#30	B	ST-213	22.14	5-5	45	18	2009
14324_4#31	B	ST-41/44	7-2.4	1-5	14	24	2009
14324_4#32	B	ST-32	7.16	3-3	1	3	2009
14324_4#33	B	ST-32	5-2.10	5-1	1	220	2009
14324_4#34	B	Other	22.9	1-5	19	17	2009
14324_4#35	B	ST-213	7-2.4	5-5	45	18	2009
14324_4#36	B	ST-32	5-2.10	5-1	1	3	2009
14324_4#37	B	ST-213	22.14	5-5	188	18	2009
14324_4#38	B	ST-41/44	7-2.4	1-5	14	2	2009
14324_4#39	B	ST-269	22.14	5-76	15	21	2009
14324_4#4	C	ST-11	5-1.10-8	3-6	902	20	2007
14324_4#40	B	ST-41/44	7-2.13-2	1-5	14	2	2009
14324_4#41	C	ST-11	5-1.10-8	3-6	-9	20	2009
14324_4#42	B	ST-41/44	7-2.4	1-5	14	24	2010
14324_4#43	B	ST-41/44	7-2.4	1-5	14	24	2010
14324_4#44	B	ST-269	19-1.15-11	3-7	15	21	2010
14324_4#45	B	ST-32	5-2.10	5-1	1	220	2010
14324_4#46	B	ST-41/44	7-2.4	1-5	14	2	2010
14324_4#47	B	ST-41/44	7-2.4	1-5	678	24	2010
14324_4#48	W/Y	ST-23	5-2.10-1	4-1	25	7	2010
14324_4#49	B	ST-41/44	17.16-93	5-5	724	10	2010

14324_4#5	B	ST-41/44	7-2.13-2	5-13	14	2	2007
14324_4#50	W135	ST-22	18-1.3	4-1	16	20	2010
14324_4#51	B	ST-41/44	7-2.13-1	1-5	14	2	2010
14324_4#52	C	ST-41/44	21-2.28	5-5	19	42	2010
14324_4#53	B	ST-32	19.13	5-1	1	3	2010
14324_4#54	B	Other	22.9	4-3	326	20	2010
14324_4#55	B	ST-213	22.14	5-5	45	18	2010
14324_4#56	B	ST-41/44	7-2.4	1-15	14	6	2010
14324_4#57	B	ST-32	5-2.10	5-1	1	3	2010
14324_4#58	B	ST-213	22.14	5-5	13	18	2011
14324_4#59	B	Other	22.9	5-12	13	17	2011
14324_4#6	C	ST-11	5-1.10-8	3-6	15	20	2007
14324_4#60	B	ST-269	5-2.10-1	5-1	210	21	2011
14324_4#61	B	ST-18	18-1.3	3-9	37	6	2011
14324_4#62	B	ST-213	22.14	5-5	191	18	2011
14324_4#63	B	ST-32	19.15	5-1	1	3	2011
14324_4#64	W/Y	ST-23	5-1.10-22	4-1	16	7	2011
14324_4#65	B	ST-41/44	21.16	1-5	14	2	2011
14324_4#66	B	ST-32	7-2.16	3-3	1	639	2011
14324_4#67	C	ST-103	18-1.3	3-9	19	24	2011
14324_4#68	B	ST-41/44	17-1.10-2	3-7	19	31	2011
14324_4#69	B	ST-32	7.16-32	3-3	1	3	2012
14324_4#7	B	ST-461	7-1.4-1	1-5	47	197	2007
14324_4#70	W/Y	ST-174	21.16-47	3-7	21	6	2012
14324_4#71	W/Y	ST-174	21.16	3-7	21	6	2012
14324_4#72	B	ST-32	5-2.10	5-1	1	3	2012
14324_4#73	B	ST-35	21-2.28	2-7	16	21	2012
14324_4#74	B	ST-1157	5.2	5-36	68	114	2012
14324_4#75	W/Y	ST-23	5-2.10-2	6-2	66	8	2012
14324_4#76	W135	ST-22	18-1.3	4-1	16	20	2012
14324_4#77	W/Y	ST-23	5-2.10-1	4-1	25	7	2012
14324_4#78	B	ST-41/44	7-2.30-2	1-5	13	2	2012
14324_4#79	B	ST-41/44	22.14	1-5	14	305	2012
14324_4#8	B	ST-32	5-2.10	5-1	-9	3	2007
14324_4#80	B	ST-32	7-2.16	3-3	1	3	2012
14324_4#81	B	ST-213	22.14	5-5	45	1216	2012
14324_4#82	B	ST-32	18-1.30	3-3	510	29	2012
14324_4#83	B	ST-213	22.14	5-5	264	18	2012
14324_4#84	W/Y	ST-23	5-2.10-1	4-1	25	7	2013
14324_4#85	B	ST-41/44	7-2.13-2	1-5	14	2	2013
14324_4#86	B	ST-269	22.9	1-55	602	17	2013
14324_4#87	B	ST-41/44	18-7.9	3-49	19	112	2013
14324_4#9	B	ST-213	22.14	5-5	275	18	2007
14324_5#1	B	Other	5-2.2-59	3-9	4	20	1985
14324_5#11	B	ST-41/44	7-2.4	1-5	14	2	1987
14324_5#13	C	ST-41/44	19.14	3-7	24	2	1987

14324_5#15	C	ST-11	5.2-1	5-5	22	29	1987
14324_5#17	C	ST-8	5-1.2-2	3-9	16	20	1988
14324_5#20	B	ST-32	19.15	5-1	1	3	1988
14324_5#22	B	ST-22	18-1.3	1-55	322	20	1988
14324_5#24	B	ST-41/44	7-2.4	1-5	14	2	1988
14324_5#27	B	ST-364	18-1.34	5-5	101	367	1988
14324_5#29	B	ST-41/44	7-1.1-17	1-7	385	-9	1989
14324_5#3	B	ST-32	19.15	5-1	1	3	1986
14324_5#32	B	ST-41/44	7-2.4	1-62	14	2	1989
14324_5#34	B	ST-41/44	7-2.4	1-5	14	2	1989
14324_5#36	C	ST-60	21.16	1-7	13	25	1989
14324_5#38	B	ST-41/44	7-2.4	1-62	14	2	1989
14324_5#41	C	ST-8	5-1.2-2	3-9	16	20	1990
14324_5#44	B	ST-8	5-2.10	3-9	16	20	1990
14324_5#46	B	ST-41/44	7-2.4	1-5	14	2	1990
14324_5#48	B	ST-269	18-1.3	1-7	4	329	1990
14324_5#5	B	ST-41/44	7-2.4	1-5	14	2	1986
14324_5#51	B	ST-41/44	7-2.4	1-5	14	2	1990
14324_5#54	B	ST-41/44	7-2.4	1-5	14	2	1990
14324_5#57	B	ST-32	7.16	3-3	1	3	1990
14324_5#60	A	ST-5	20.9	3-1	5	27	1990
14324_5#62	C	ST-11	5.2-1	5-5	22	29	1990
14324_5#64	C	ST-32	7-2.16	3-3	1	3	1990
14324_5#67	B	ST-41/44	7.2-13-2	1-5	14	2	1990
14324_5#7	B	ST-41/44	7-2.4	1-62	14	2	1986
14324_5#70	C	ST-8	5-1.2	3-9	16	89	1991
14324_5#72	C	ST-8	21.16	3-9	16	20	1991
14324_5#74	B	ST-41/44	-9	3-9	19	43	1991
14324_5#76	C	ST-11	5.2	3-6	128	20	1991
14324_5#79	B	Other	-9	4-3	94	21	1991
14324_5#81	B	ST-41/44	7-2.4	1-5	14	2	1991
14324_5#83	B	ST-8	5-2.10	3-9	16	20	1991
14324_5#85	C	ST-11	5.2	3-6	128	20	1991
14324_5#87	B	ST-41/44	7-2.4	1-5	14	2	1991
14324_5#9	W135	ST-22	18-1.3	1-24	24	20	1986
14324_5#90	C	ST-11	5.2	3-6	128	20	1992
14324_5#92	B	ST-41/44	7-2.4	1-5	14	2	1992
14324_5#95	B	ST-8	5-2.10	3-9	16	20	1992
14355_5#1	B	ST-32	7.16-6	3-3	1	3	2000
14355_5#10	B	ST-41/44	7-2.4	1-5	14	2	2000
14355_5#11	B	ST-41/44	7-2.13-2	1-5	14	2	2000
14355_5#12	B	ST-41/44	7-2.4	1-5	14	2	2000
14355_5#13	B	ST-41/44	7-2.4	1-5	14	2	2000
14355_5#14	B	ST-32	7-4.4-1	5-1	1	3	2000
14355_5#15	B	ST-41/44	7-2.13-2	1-5	14	2	2000
14355_5#16	C	ST-11	5-1.10-8	5-1	10	20	2000

14355_5#17	B	ST-32	7-2.16	3-3	1	94	2000
14355_5#18	B	ST-32	5-2.10	5-1	1	3	2000
14355_5#19	B	ST-41/44	18-7.9	1-5	14	2	2000
14355_5#2	B	ST-32	5-2.10	5-1	1	3	2000
14355_5#20	B	ST-41/44	7-2.4	1-5	14	2	2000
14355_5#21	B	ST-32	5-2.10	5-1	1	3	2000
14355_5#22	B	ST-41/44	7-2.4	1-5	14	2	2000
14355_5#23	B	ST-41/44	7-2.13-2	1-5	14	2	2000
14355_5#24	B	ST-41/44	7-2.4	1-5	14	2	2000
14355_5#25	C	ST-11	5-1.10-8	3-6	10	20	2000
14355_5#26	C	ST-11	5.2	1-1	129	20	2000
14355_5#27	B	ST-41/44	7-2.13-2	1-5	14	2	2000
14355_5#28	B	ST-41/44	7-2.13-2	1-5	14	2	2001
14355_5#29	C	ST-11	5-1.10-8	3-6	10	20	2001
14355_5#3	B	ST-461	19-2.13-1	3-9	47	118	2000
14355_5#30	B	ST-41/44	7-2.13-2	4-1	14	2	2001
14355_5#31	B	ST-32	5-2.10	5-1	1	3	2001
14355_5#32	B	ST-269	5.2	5-1	15	21	2001
14355_5#33	C	ST-11	5.2	3-6	131	20	2001
14355_5#34	C	ST-8	5.2	3-9	16	20	2001
14355_5#35	B	ST-41/44	7-2.4	1-5	14	305	2001
14355_5#36	B	ST-41/44	7-2.4	1-5	14	2	2001
14355_5#37	B	ST-41/44	7-2.4	1-5	14	2	2001
14355_5#38	C	ST-11	5-1.10-1	3-6	10	20	2001
14355_5#39	B	ST-41/44	7-2.4	1-5	14	2	2001
14355_5#4	C	ST-11	5-1.10-8	3-6	10	20	2000
14355_5#40	B	ST-269	22.9	5-12	61	17	2001
14355_5#41	C	ST-18	5-1.10-27	3-6	37	6	2001
14355_5#42	B	ST-41/44	7-2.4	1-5	14	2	2001
14355_5#43	B	ST-41/44	7-2.4	1-5	14	305	2001
14355_5#44	C	ST-11	5.2	1-1	129	20	2001
14355_5#45	C	ST-11	5.2	3-6	131	20	2001
14355_5#46	C	ST-11	5-1.10-8	5-5	319	20	2001
14355_5#47	B	ST-32	7.16	3-3	127	3	2001
14355_5#48	B	ST-41/44	7-2.4	1-5	14	2	2001
14355_5#49	B	ST-41/44	7-2.4	1-5	14	2	2001
14355_5#5	C	ST-461	19.13-2	3-9	125	197	2000
14355_5#50	B	ST-41/44	7-2.4	1-5	14	9	2001
14355_5#51	B	ST-32	5-2.10-31	5-1	1	3	2002
14355_5#52	B	ST-41/44	7-2.4	1-5	14	2	2002
14355_5#53	B	ST-41/44	7-2.4	1-5	14	305	2002
14355_5#54	C	ST-11	5.2	3-6	128	1135	2002
14355_5#55	B	ST-32	5-2.10	5-1	1	3	2010
14355_5#56	B	ST-41/44	7-2.13-2	1-5	14	2	2010
14355_5#57	B	ST-41/44	7-2.4	1-5	14	2	2010
14355_5#58	B	ST-41/44	7-2.4	1-5	14	2	2010

14355_5#59	C	ST-8	5.2	3-9	16	20	2010
14355_5#6	B	ST-32	7.1	3-3	-9	3	2000
14355_5#60	C	ST-11	5.2	1-1	129	20	2010
14355_5#61	B	ST-41/44	21.16	1-5	14	43	2010
14355_5#62	C	ST-8	5.2	3-9	14	20	2010
14355_5#63	B	ST-269	19-1.14-6	5-1	15	21	2010
14355_5#64	B	ST-41/44	7-2.4	4-1	4	2	2010
14355_5#65	B	ST-32	5-2.4	5-1	1	3	2010
14355_5#66	B	ST-41/44	7-2.4	1-5	14	305	2010
14355_5#67	B	ST-41/44	7-2.4	1-5	14	2	2010
14355_5#68	B	ST-60	18-1.1	5-8	13	25	2010
14355_5#69	C	ST-11	5-1.10-4	3-6	4	20	2010
14355_5#7	B	ST-41/44	7-2.9	1-5	120	2	2000
14355_5#70	B	ST-41/44	7-2.4	3-5	14	2	2010
14355_5#71	B	ST-32	5-2.10	5-1	1	3	2010
14355_5#72	C	ST-11	5.2	1-1	129	20	2010
14355_5#73	B	ST-41/44	7-2.4	5-2	14	2	2010
14355_5#74	C	ST-11	5.2	1-1	129	20	2010
14355_5#75	B	ST-60	7-2.4	5-7	13	24	2010
14355_5#76	B	ST-18	17.16-4	5-2	37	6	2010
14355_5#77	C	ST-11	5.2	1-1	129	20	2010
14355_5#78	B	Other	21.16	5-2	320	306	2010
14355_5#79	B	ST-41/44	7-2.13-1	1-5	14	2	2011
14355_5#8	B	ST-269	19-1.14-6	5-1	31	21	2000
14355_5#80	B	ST-41/44	7-2.4	1-18	14	2	2011
14355_5#81	C	ST-11	5-1.10-8	3-6	10	20	2011
14355_5#82	C	ST-8	5.2	3-9	16	20	2011
14355_5#83	B	Other	22-1.14	1-25	13	24	2011
14355_5#84	B	ST-41/44	7-2.13-1	1-7	283	2	2011
14355_5#85	C	ST-11	5-1.10-8	3-6	130	20	2011
14355_5#86	C	ST-11	5-1.10-8	3-6	10	20	2011
14355_5#87	C	ST-11	5.2	3-6	89	20	2011
14355_5#88	B	ST-32	7.16-32	3-3	1	3	2011
14355_5#89	B	ST-32	7.13-2	3-3	1	3	2011
14355_5#9	B	ST-41/44	18-7.9	1-5	14	2	2000
14355_5#90	B	ST-41/44	7-2.4	1-5	4	2	2011
14355_5#91	C	ST-11	5.2	1-1	129	20	2011
14355_5#92	B	ST-32	7.16	3-3	1	3	2011
14355_5#93	C	ST-11	5.2	3-6	131	20	2011
14355_5#94	B	ST-32	19.15	5-1	1	3	2011
14355_5#95	B	ST-269	19-1.14-6	5-1	15	21	2011
14355_6#1	C	ST-11	5-1.10-8	3-6	10	20	2011
14355_6#10	B	ST-32	19.15	5-1	1	3	2011
14355_6#11	B	ST-41/44	7-2.4	1-5	11	2	2012
14355_6#12	B	ST-32	5-2.10	5-1	1	220	2012
14355_6#13	C	ST-11	5.2	3-6	126	20	2012

14355_6#14	C	ST-11	5.2	1-116	89	20	2012
14355_6#15	C	ST-269	5-1.2-2	5-1	15	21	2012
14355_6#16	B	Other	7-2.4	1-5	14	2	2012
14355_6#17	C	ST-11	5-1.10-8	3-6	10	20	2012
14355_6#18	C	ST-8	5.2	3-9	16	20	2012
14355_6#19	C	ST-11	5-1.10-8	5-13	107	20	2012
14355_6#2	B	ST-32	7-1.4-1	5-5	1	3	2011
14355_6#20	C	ST-11	7-2.16	3-6	118	20	2012
14355_6#21	C	ST-11	5.2	1-1	129	20	2012
14355_6#22	B	ST-41/44	7-2.4	1-5	14	2	2012
14355_6#23	C	ST-11	5.2	1-1	129	20	2012
14355_6#24	C	ST-11	-9	3-6	10	20	2012
14355_6#25	C	ST-11	5-1.10-8	3-6	10	20	2002
14355_6#27	B	ST-60	5.2	1-7	13	24	2002
14355_6#28	C	ST-11	5-1.10-8	3-6	10	20	2002
14355_6#29	C	ST-8	5.2	3-9	16	20	2002
14355_6#3	B	ST-41/44	7.30-2	1-5	14	2	2011
14355_6#30	C	ST-11	5-1.10-8	3-6	121	20	2002
14355_6#31	W/Y	ST-174	21.16	3-7	21	6	2002
14355_6#32	C	ST-11	5-1.10-8	3-6	10	20	2002
14355_6#33	C	ST-11	5-1.10-8	3-6	10	20	2002
14355_6#34	B	ST-41/44	22-1.14	1-5	14	2	2002
14355_6#35	C	ST-11	21.16	1-30	110	29	2002
14355_6#36	C	ST-11	5.2	5-5	25	29	2002
14355_6#37	B	ST-213	22.14	1-7	187	18	2002
14355_6#38	C	ST-11	5.2	1-1	129	20	2002
14355_6#39	C	ST-11	5-1.10-8	3-49	10	20	2002
14355_6#4	C	ST-11	5-1.10-8	5-1	-9	20	2011
14355_6#40	C	ST-11	5.2	1-1	129	20	2002
14355_6#41	C	ST-11	5-1.10-8	3-6	10	20	2002
14355_6#42	C	ST-11	5-1.10-8	3-6	10	20	2002
14355_6#43	B	ST-41/44	7-2.4	1-5	14	2	2002
14355_6#44	C	ST-11	5.2	1-1	129	20	2002
14355_6#45	C	ST-11	5-1.10-8	3-6	10	20	2002
14355_6#46	B	ST-41/44	7-2.13-2	4-1	14	2	2002
14355_6#47	B	ST-364	-9	1-20	119	6	2002
14355_6#48	C	ST-11	5-1.10-8	3-6	10	20	2002
14355_6#49	B	ST-41/44	5-1.10-8	1-7	13	29	2002
14355_6#5	C	ST-11	5-1.10-8	3-6	10	20	2011
14355_6#50	C	ST-8	5.2	1-7	16	20	2002
14355_6#51	C	ST-8	5.2	3-9	16	20	2002
14355_6#52	B	ST-41/44	31.16	1-5	72	2	2002
14355_6#53	B	ST-269	22.9	5-12	19	17	2002
14355_6#54	B	ST-35	22-1.14	4-1	16	21	2002
14355_6#55	B	ST-41/44	7-2.4	1-5	4	2	2002
14355_6#56	W135	ST-22	18.25	4-1	16	20	2006

14355_6#57	B	ST-41/44	7-2.4	1-5	14	9	2006
14355_6#58	B	ST-41/44	7-2.4	3-5	14	2	2006
14355_6#59	B	ST-32	5-2.10	5-1	1	3	2006
14355_6#6	C	ST-11	5-1.10-8	3-6	10	20	2011
14355_6#60	B	ST-41/44	7-2.4	1-5	4	2	2006
14355_6#61	B	ST-41/44	7-2.4	1-5	14	2	2006
14355_6#62	B	ST-22	18-1.3	1-5	16	20	2006
14355_6#63	B	ST-269	19-1.15-11	1-7	15	21	2006
14355_6#64	B	ST-32	5-2.10	5-50	1	3	2006
14355_6#65	B	ST-32	5-2.10	5-1	1	3	2006
14355_6#66	C	ST-11	5.2	3-6	433	20	2006
14355_6#68	B	ST-269	19-1.15-11	3-7	15	21	2006
14355_6#69	B	ST-41/44	7-2.4	1-5	14	2	2006
14355_6#7	C	ST-269	5-1.10-1	4-1	-9	2	2011
14355_6#70	B	ST-32	18-1.30-1	3-3	510	29	2006
14355_6#71	W/Y	ST-167	5-1.10-4	5-2	23	9	2006
14355_6#72	B	ST-41/44	7-2.4	5-5	4	2	2006
14355_6#73	B	ST-32	7-2.16	3-3	1	3	2006
14355_6#74	W/Y	ST-167	5-1.10-4	4-1	23	1214	2007
14355_6#75	B	ST-41/44	7-2.4	1-5	14	2	2007
14355_6#76	B	ST-32	5-2.10	5-1	1	3	2007
14355_6#77	B	Other	7-2.4	1-5	4	2	2007
14355_6#78	B	ST-41/44	7-2.4	3-6	123	2	2007
14355_6#79	W/Y	ST-174	21.16	3-7	21	6	2007
14355_6#8	B	ST-41/44	7-1.1	1-5	14	129	2011
14355_6#80	W135	ST-22	18-1.3	4-1	110	20	2007
14355_6#81	B	Other	5-1.10-1	1-5	16	24	2007
14355_6#82	B	ST-269	22.9	5-12	13	17	2007
14355_6#83	B	ST-41/44	18-1.3	1-5	14	47	2007
14355_6#84	B	ST-269	19-1.15-11	3-7	15	21	2007
14355_6#85	29E	ST-60	5.2	1-7	144	24	2007
14355_6#86	B	ST-41/44	7-2.13-2	4-1	14	2	2007
14355_6#87	X	Other	5-1.2-2	5-1	12	12	2007
14355_6#88	B	ST-41/44	18-7.9	1-7	19	112	2007
14355_6#89	B	ST-41/44	7-2.13-2	1-7	14	-9	2007
14355_6#9	B	ST-32	7-2.16	3-49	1	94	2011
14355_6#90	B	ST-32	7.16	3-3	583	3	2007
14355_6#91	C	ST-8	5.2	5-5	16	20	2007
14355_6#92	B	ST-213	22.14-3	5-5	45	18	2007
14355_6#93	B	ST-269	22.9	5-12	4	17	2007
14355_6#94	B	ST-41/44	7-2.4	3-1	14	9	2007
14355_6#95	B	ST-32	7.16	3-3	1	3	2007
14355_7#11	C	ST-11	5.2	3-6	128	20	1992
14355_7#13	B	ST-41/44	7-2.4	1-5	14	2	1992
14355_7#15	B	ST-41/44	7-2.4	1-5	14	2	1992
14355_7#18	B	ST-41/44	18.4	1-5	14	2	1992

14355_7#20	B	ST-18	5-1.2-2	1-5	59	-9	1992
14355_7#22	B	ST-32	7.16	3-3	1	3	1993
14355_7#25	C	ST-11	5.2	4-1	459	20	1993
14355_7#29	B	ST-32	19.15	5-1	1	3	1993
14355_7#3	B	ST-32	19.15	5-1	1	3	1992
14355_7#31	B	ST-269	7-2.14	4-3	4	21	1993
14355_7#35	B	ST-41/44	7-2.4	1-5	14	659	1993
14355_7#38	C	ST-11	5.2-1	5-5	318	29	1994
14355_7#40	B	ST-8	5-2.10	3-9	71	20	1994
14355_7#41	B	ST-8	-9	3-9	71	20	1994
14355_7#43	B	ST-41/44	7-2.4	1-5	19	337	1994
14355_7#45	B	ST-32	5-2.10	5-1	1	3	1994
14355_7#47	B	ST-32	7.16	3-3	1	3	1994
14355_7#50	C	ST-41/44	18-3.1	5-2	19	1	1994
14355_7#53	B	ST-41/44	7-2.4	1-5	14	2	1995
14355_7#56	B	ST-41/44	18-1.3	1-5	29	2	1995
14355_7#58	B	ST-41/44	7-2.4	1-5	14	2	1995
14355_7#6	C	ST-11	5.2	3-6	128	20	1992
14355_7#61	B	ST-35	22-1.14	5-5	14	24	1995
14355_7#63	B	ST-41/44	7-2.4	1-5	14	2	1996
14355_7#66	B	Other	5-2.10	5-5	735	24	1996
14355_7#68	B	ST-41/44	7-2.4	5-7	14	2	1996
14355_7#70	B	ST-32	5-2.10	5-1	1	3	1997
14355_7#72	B	ST-41/44	18-1.34	1-5	19	890	1997
14355_7#74	B	ST-41/44	7-2.4	1-5	14	2	1997
14355_7#77	B	ST-32	5-2.10-62	5-1	-9	3	1997
14355_7#79	C	ST-11	5-1.10-8	4-1	823	20	1997
14355_7#8	C	ST-11	5.2	3-6	128	20	1997
14355_7#81	B	ST-213	22.14	5-5	110	18	1997
14355_7#83	B	ST-32	7.16	3-3	1	3	1998
14355_7#85	B	ST-41/44	7-2.15	4-1	14	2	1998
14355_7#88	B	ST-41/44	7-2.4	1-5	14	2	1998
14355_7#91	B	ST-41/44	-9	1-5	14	2	1998
14355_7#94	B	ST-41/44	7-2.4	1-5	14	2	1998
14355_8#11	B	ST-41/44	7-2.4	1-5	14	26	1999
14355_8#14	B	ST-41/44	7-2.4	1-7	123	2	1999
14355_8#16	B	ST-41/44	7-2.4	1-5	14	2	1999
14355_8#19	B	ST-41/44	7-2.2	1-5	14	2	2000
14355_8#2	B	ST-41/44	7-2.4	1-5	14	2	1998
14355_8#22	B	ST-41/44	7-2.13-2	3-16	14	2	2010
14355_8#24	B	ST-41/44	7-2.13-2	1-5	14	2	2010
14355_8#26	B	ST-32	7.16	3-3	1	3	2011
14355_8#29	B	ST-41/44	7-2.4	1-5	14	2	2012
14355_8#31	B	ST-41/44	18-7.9	1-7	19	112	2003
14355_8#33	B	ST-213	22.14	5-5	191	18	2003
14355_8#5	B	ST-41/44	18-1.3	1-5	174	24	1998

14355_8#8	B	ST-41/44	22.14-6	1-7	19	43	1999
14555_6#51	B	ST-41/44	7-2.13-2	1-5	14	2	1997
14555_6#52	B	ST-41/44	7-2.4	1-5	322	2	1997
14555_6#53	B	ST-41/44	7-2.4	1-5	14	2	1997
14555_6#54	B	ST-41/44	5-1.10-8	1-7	19	112	1997
14555_6#55	B	ST-41/44	21.16	1-5	14	2	1997
14555_6#57	B	ST-41/44	19.15	1-5	14	-9	1998
14555_6#58	B	ST-41/44	7-2.4	1-5	14	2	1998
14555_6#60	C	ST-11	5-1.10-4	4-1	4	20	1999
14555_6#62	B	ST-41/44	7-2.4	1-5	14	2	1999
14555_6#63	B	ST-32	7.16-32	3-49	1	3	2000
14555_6#64	B	ST-8	5-1.2-2	3-6	16	20	1978
14555_6#66	A	ST-1	5-2.10	5-1	4	29	1979
14555_6#68	B	ST-8	5-1.2-2	5-99	16	20	1979
14555_6#71	B	ST-8	5-1.2-2	3-9	16	20	1979
14555_6#74	B	ST-8	-9	1-1	944	129	1980
14555_6#76	B	ST-8	5-9.10	1-5	16	20	1981
14555_6#79	B	ST-41/44	7-2.4	1-5	14	2	1982
14555_6#81	W/Y	ST-23	5-1.2-2	5-8	25	6	1983
14555_6#84	C	Other	-9	3-9	16	20	1984
14555_6#86	B	ST-269	12-1.16-8	5-2	106	21	1984
14555_6#88	B	ST-41/44	7-2.4	1-5	14	2	1984
14555_6#91	B	Other	5-4.2	5-5	22	-9	1985
14555_6#93	W135	ST-11	5.2	1-1	22	29	1985
14672_1#1	B	ST-41/44	7-2.4	4-3	123	2	1998
14672_1#10	B	ST-41/44	7-2.4	1-5	14	2	1998
14672_1#11	B	ST-41/44	7-2.4	1-5	14	2	1998
14672_1#12	B	ST-269	19-1.14-6	5-1	15	21	1998
14672_1#13	B	ST-41/44	7-2.13-2	1-5	14	2	1998
14672_1#14	B	ST-41/44	7-2.4	1-5	14	2	1998
14672_1#15	B	ST-41/44	7-2.4	1-5	14	2	1998
14672_1#16	B	ST-269	19-1.14-6	5-1	15	21	1998
14672_1#17	B	ST-41/44	7-2.4	4-3	123	2	1998
14672_1#18	B	ST-269	-9	5-12	19	17	1999
14672_1#19	B	ST-32	7.16	3-3	1	3	1999
14672_1#2	C	ST-60	5.2	3-16	13	24	1998
14672_1#20	B	ST-41/44	7-1.1	1-5	14	2	1999
14672_1#21	C	ST-11	5.2	5-5	22	29	1999
14672_1#22	B	ST-41/44	7-2.4	1-5	14	-9	1999
14672_1#23	B	ST-41/44	7-2.13-2	1-5	14	2	1999
14672_1#24	B	ST-41/44	7-2.13-2	1-5	14	2	1999
14672_1#25	B	ST-41/44	7-2.4	1-5	14	2	1999
14672_1#26	B	ST-41/44	7-2.13-9	4-1	14	24	1999
14672_1#27	B	ST-32	-9	5-1	1	3	1999
14672_1#28	B	ST-41/44	-9	1-5	14	2	1999
14672_1#29	B	ST-41/44	7-2.4	1-5	14	2	1999

14672_1#3	B	ST-41/44	7-2.4	1-5	14	2	1998
14672_1#30	B	ST-41/44	7-2.4-13	1-5	14	2	1999
14672_1#31	B	ST-41/44	7-2.4	1-5	14	2	1999
14672_1#32	B	ST-41/44	7-2.4	1-5	14	58	1999
14672_1#33	B	ST-41/44	21.16	1-7	19	-9	1999
14672_1#34	B	ST-32	7.16	3-3	1	3	1999
14672_1#35	B	ST-41/44	7-2.4	1-5	4	145	1999
14672_1#36	B	ST-41/44	18-7.9	1-7	19	112	1999
14672_1#37	B	ST-212	19-3.15	5-8	19	47	1999
14672_1#38	C	ST-11	5.2-3	5-5	25	29	1999
14672_1#39	W135	ST-22	18-1.3	4-1	16	20	1999
14672_1#4	B	ST-32	5-2.10	5-1	1	3	1998
14672_1#40	B	ST-32	7.16	3-3	1	3	1999
14672_1#41	C	ST-11	5.2	5-5	22	29	1999
14672_1#42	C	ST-11	5.2-3	5-5	25	29	1999
14672_1#43	B	ST-32	7.16	3-3	1	3	1999
14672_1#44	B	ST-41/44	7-2.4	1-5	14	2	1999
14672_1#45	B	ST-32	7-2.16	3-3	1	3	1999
14672_1#46	C	ST-11	5-1.10-8	3-6	10	20	1999
14672_1#47	B	ST-269	-9	5-1	15	21	1999
14672_1#48	B	ST-32	5-7.10	5-1	1	3	1999
14672_1#5	B	ST-41/44	7-2.4	1-5	14	2	1998
14672_1#50	B	ST-41/44	7-2.4	1-5	14	2	1999
14672_1#51	B	ST-32	7.16	3-3	1	3	1999
14672_1#52	B	ST-32	5-2.10	5-1	1	3	1999
14672_1#53	B	ST-32	5-2.10	5-1	1	3	1999
14672_1#54	B	ST-32	5-2.10	5-1	1	3	1999
14672_1#55	C	ST-11	5.2	1-5	19	20	1999
14672_1#56	B	ST-41/44	7-2.4	1-5	14	2	1999
14672_1#57	B	ST-269	19-1.15-11	5-1	15	21	1999
14672_1#58	C	ST-8	5.2	3-9	16	20	1999
14672_1#59	B	ST-41/44	7-2.13-2	1-5	14	2	1999
14672_1#6	B	ST-41/44	7-2.4	1-5	14	2	1998
14672_1#60	B	ST-32	7-2.16-66	3-3	1	94	1999
14672_1#61	B	ST-41/44	5-2.10-2	1-5	14	2	1999
14672_1#62	C	ST-11	21.16	1-30	22	29	1999
14672_1#63	B	ST-41/44	17.16-3	1-5	24	43	1999
14672_1#64	B	ST-41/44	7-2.13-2	1-7	14	101	1999
14672_1#65	B	ST-41/44	7-2.4	1-5	14	13	1999
14672_1#66	W/Y	ST-167	5-1.10-4	4-1	23	9	1999
14672_1#67	B	ST-41/44	7-2.13-2	1-22	14	2	1999
14672_1#68	B	ST-32	19.15	5-1	1	3	1999
14672_1#69	B	ST-41/44	7-2.4	1-5	14	2	1999
14672_1#7	B	ST-41/44	7-2.4-31	1-5	14	2	1998
14672_1#71	B	ST-41/44	7-2.4	1-5	14	9	1999
14672_1#72	B	Other	7-1.1	5-1	4	122	1999

14672_1#74	B	ST-41/44	7.2-13-2	1-5	14	2	1999
14672_1#75	B	ST-41/44	7-2.4	1-5	14	2	1999
14672_1#76	C	ST-11	21.16	1-30	22	29	1999
14672_1#77	C	ST-11	-9	3-6	10	20	1999
14672_1#78	B	ST-41/44	22.14-6	1-7	19	43	1999
14672_1#79	C	ST-11	5-1.10-8	3-6	10	20	1999
14672_1#8	B	ST-41/44	7.2-13-2	4-1	14	2	1998
14672_1#80	W/Y	ST-92	5-1.10-10	4-3	21	9	1999
14672_1#81	C	ST-11	5-1.10-8	3-6	78	20	1999
14672_1#82	B	ST-41/44	7-2.4	1-5	4	2	2000
14672_1#83	B	ST-32	7.16-32	3-3	1	3	2000
14672_1#84	B	ST-41/44	7-2.4	1-5	14	2	2000
14672_1#85	B	ST-41/44	7-2.4	1-5	122	2	2000
14672_1#86	B	ST-41/44	7-2.4	1-5	14	2	2000
14672_1#87	B	ST-41/44	7-2.4	1-80	14	2	2000
14672_1#88	B	ST-269	5.2	5-1	15	21	2000
14672_1#89	B	ST-41/44	7-2.4	1-7	14	2	2000
14672_1#9	B	ST-41/44	7-2.4	1-5	14	2	1998
14672_1#90	B	ST-269	7.16	5-1	15	21	2000
14672_1#91	C	ST-11	5-1.10-8	5-5	319	20	2000
14672_1#92	B	ST-32	19.15	5-1	1	3	2000
14672_1#93	B	ST-41/44	7-2.4	1-28	14	2	2000
14672_1#94	B	ST-41/44	7-2.4	1-5	14	2	2000
14672_1#95	B	ST-41/44	7-2.13-2	1-5	14	13	2000
14893_3#67	C	ST-11	-9	5-5	22	29	1999
14893_3#68	B	ST-32	7-2.16	3-3	1	94	1999
14893_3#69	B	ST-269	19-1.15-11	5-1	15	18	2002
14893_3#70	B	ST-32	5-2.10	5-1	1	3	2006

Supplementary Table S3.

Distribution of PorA, FetA, NHBA and FHbp major antigen alleles over sequences in the ST-41/44 sub-lineage

Peptide allele	ST-41/44 sub-lineage	Not in ST-41/44 sub-lineage
PorA		
7-2.4	121	2
other	57	306
FetA		
1-5	146	12
other	32	296
NHBA		
2	149	3
other	29	305
FHbp		
14	153	4
other	25	304

Supplementary Table S4.

Ratio of non-synonymous versus synonymous mutations in all sequences, serogroup B or major clusters in serogroup B isolates

dN/dS	fetA	fHbp	NHBA	porA	Average
All sequences	0.3357	0.4347	0.5934	0.6235	0.4971
Serogroup B	0.3303	0.4503	0.5886	0.6485	0.5044
ST-41/44 sub-lineage	0.3400	0.4113	0.5777	0.6433	0.4931
ST-32 complex	0.3518	0.3853	0.6334	0.7361	0.5267
ST-41/44 sub-lineage late period	0.3183	0.4178	0.5908	0.7259	0.5132
ST-41/44 excluding sub-lineage	0.3410	0.3862	0.5787	0.5868	0.4732

Supplementary Figure S1.

Pan genome (total) = 7530 gene groups

Core genome (99-100% strains) = 1175 gene groups

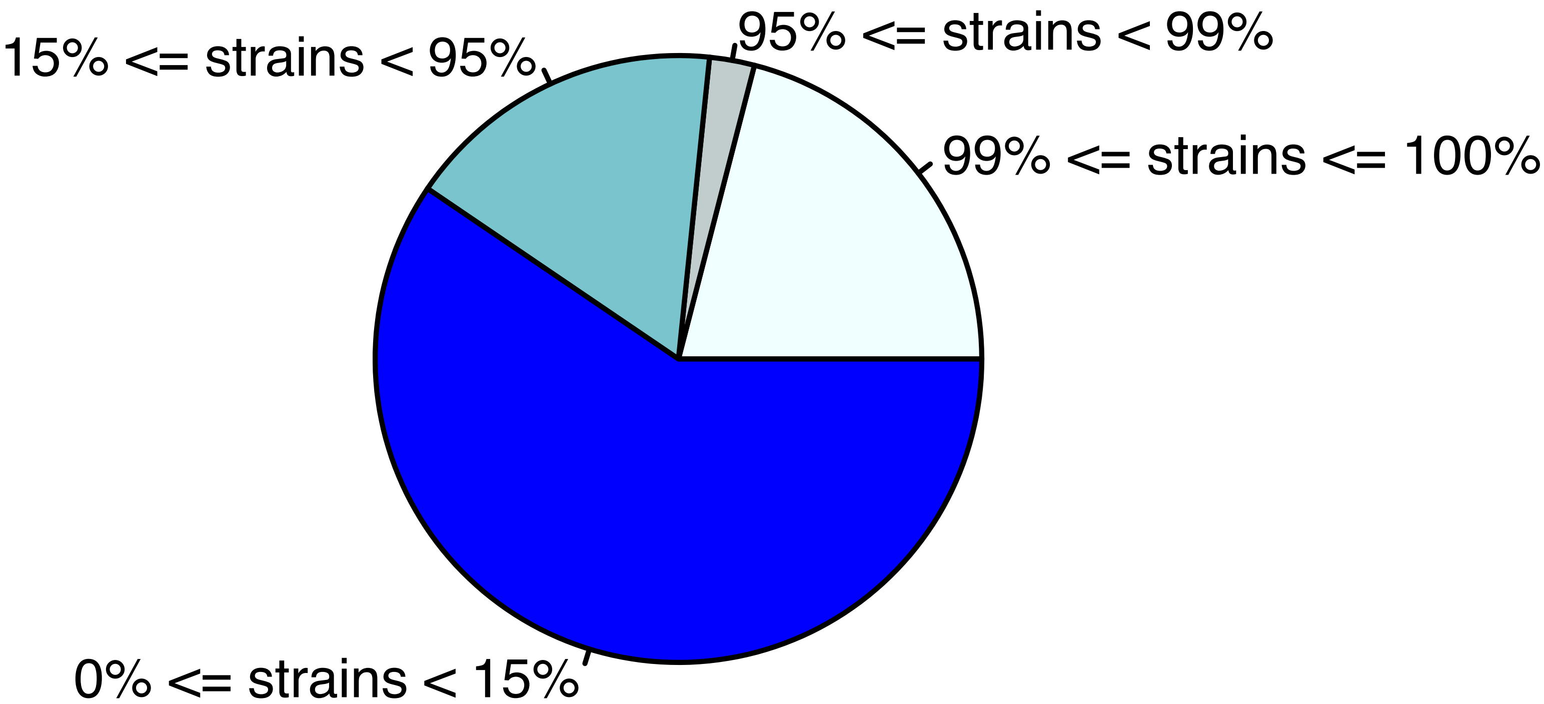

Supplementary Figure S2.

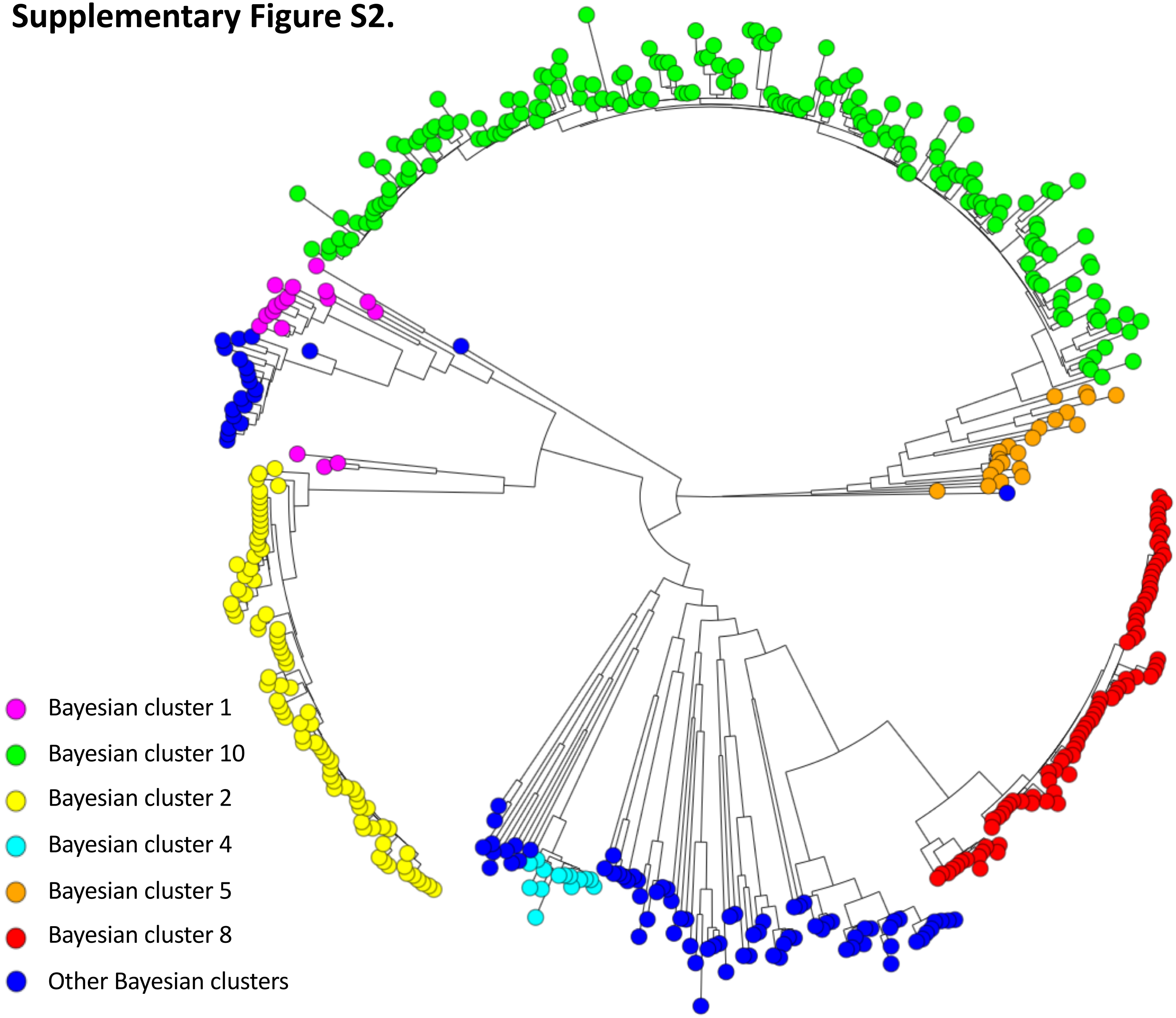

Supplementary Figure S3.

ST-41/44 sub-lineage, MRCA 56 years (95% CI 47 – 74 years) ago

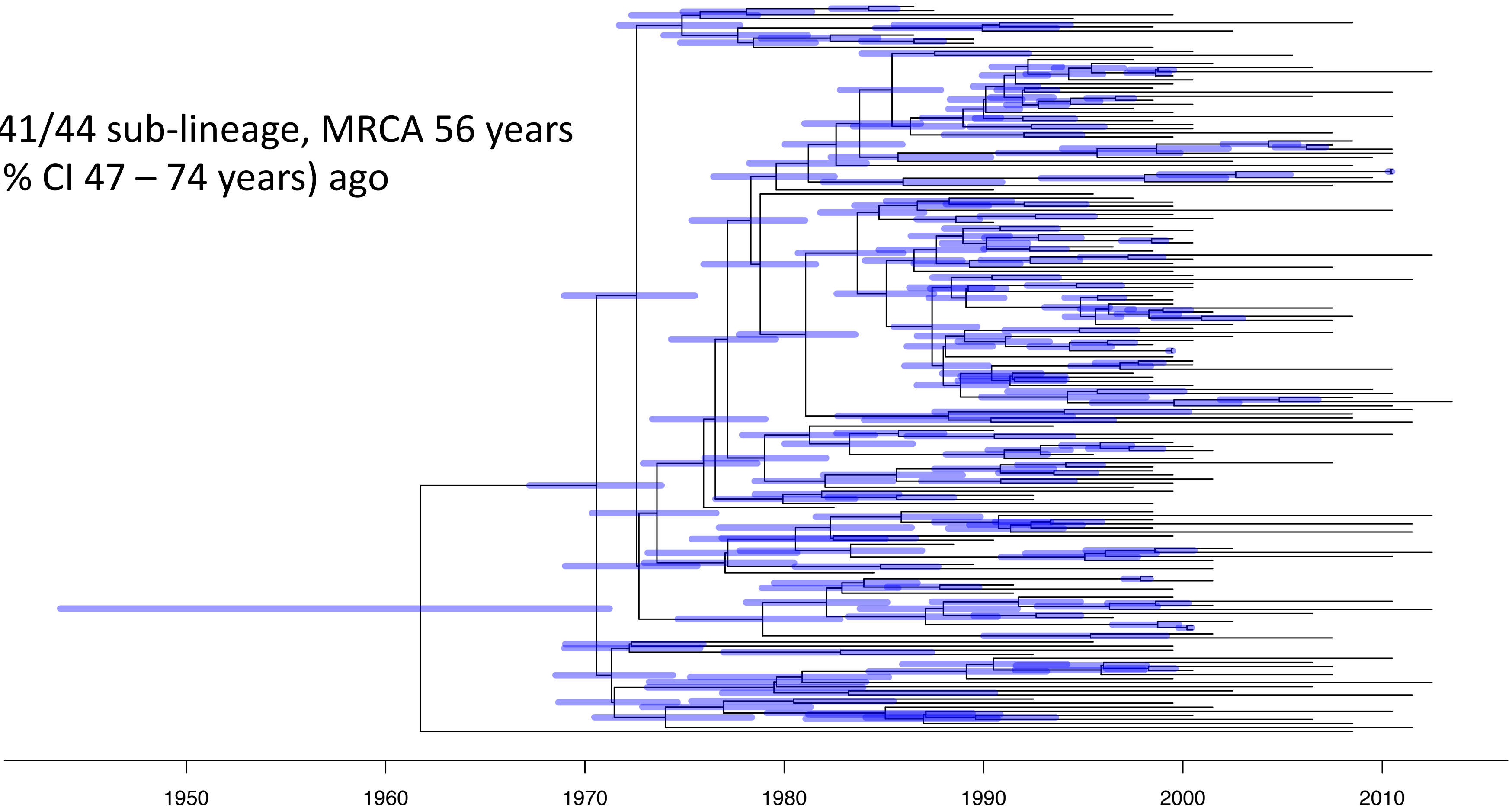

Supplementary Figure S4.

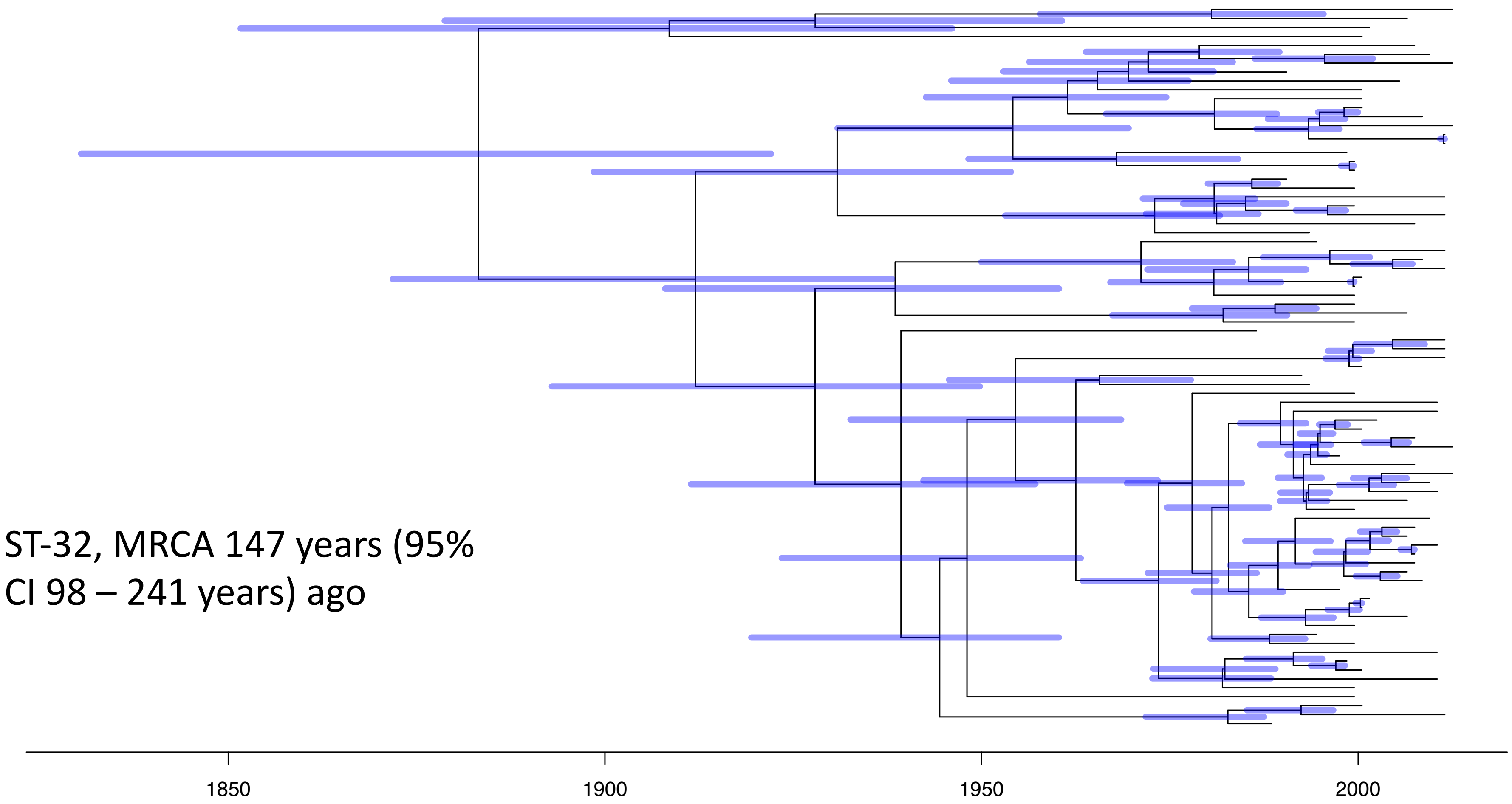

Supplement: Supplementary material 1 [file mgen-6-422-s001.pdf]
